# Is Cu_3–*x*_P a Semiconductor, a Metal, or a Semimetal?

**DOI:** 10.1021/acs.chemmater.2c03283

**Published:** 2023-01-25

**Authors:** Andrea Crovetto, Thomas Unold, Andriy Zakutayev

**Affiliations:** † Materials Science Center, National Renewable Energy Laboratory, Golden, Colorado80401, United States; ‡ Department of Structure and Dynamics of Energy Materials, Helmholtz-Zentrum Berlin für Materialien und Energie GmbH, 14109Berlin, Germany; ¶ National Centre for Nano Fabrication and Characterization (DTU Nanolab), Technical University of Denmark, 2800Kongens Lyngby, Denmark

## Abstract

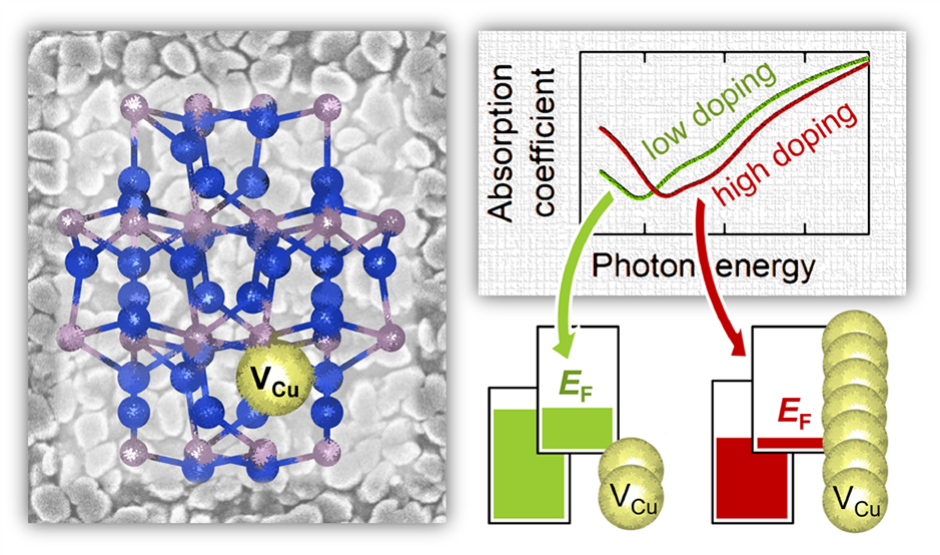

Despite the recent surge in interest in Cu_3–*x*_P for catalysis, batteries, and plasmonics, the electronic nature of Cu_3–*x*_P remains unclear. Some studies have shown evidence of semiconducting behavior, whereas others have argued that Cu_3–*x*_P is a metallic compound. Here, we attempt to resolve this dilemma on the basis of combinatorial thin-film experiments, electronic structure calculations, and semiclassical Boltzmann transport theory. We find strong evidence that stoichiometric, defect-free Cu_3_P is an intrinsic semimetal, i.e., a material with a small overlap between the valence and the conduction band. On the other hand, experimentally realizable Cu_3–*x*_P films are always p-type semimetals natively doped by copper vacancies regardless of *x*. It is not implausible that Cu_3–*x*_P samples with very small characteristic sizes (such as small nanoparticles) are semiconductors due to quantum confinement effects that result in the opening of a band gap. We observe high hole mobilities (276 cm^2^/(V s)) in Cu_3–*x*_P films at low temperatures, pointing to low ionized impurity scattering rates in spite of a high doping density. We report an optical effect equivalent to the Burstein–Moss shift, and we assign an infrared absorption peak to bulk interband transitions rather than to a surface plasmon resonance. From a materials processing perspective, this study demonstrates the suitability of reactive sputter deposition for detailed high-throughput studies of emerging metal phosphides.

## Introduction

Most binary compounds consisting of Cu(I) and a moderately electronegative element are interesting semiconductors for optoelectronics and/or thermoelectrics. Cu_2_O-based solar cells have reached photovoltaic efficiencies close to 10%.^1^ Cu_2_O itself is the host of the largest excitonic wave functions ever discovered.^2^ Cu_2_S was one of the prominent thin-film solar cell materials in the 1970s^3^ and later regained prominence in nanoparticle form as a semiconductor system with localized surface plasmon resonances in the near-infrared.^4^ Cu_2_Se and Cu_2_Te are among the best p-type thermoelectric materials known today,^5,6^ with record *zT* values above 1 at high temperatures. CuI is currently the p-type transparent conductor with the highest figure of merit^7,8^ and one of the highest-performing transparent thermoelectric materials.^9^ Cu_3_N is a defect-tolerant semiconductor^10^ with an ideal band gap for photovoltaics.^11^ CuP_2_ is an unconventional semiconductor containing P–P bonds, with optimal band gap and doping density for photovoltaics.^12^

Cu_3_P is another copper(I) phosphide, which has been synthesized in the form of single crystals,^13,14^ powders,^15−18^ thin films,^19−23^ and especially nanoparticles and other nanostructured forms.^24−30^ Cu_3_P is usually found to be strongly p-type due to substantial Cu deficiency^13,17^ and is therefore often referred to as Cu_3–*x*_P. In recent years, Cu_3–*x*_P has been the subject of intense research as an electro- and photocatalyst for various reactions,^20,21,25,27,28,30^ as well as a battery anode.^31,32^ However, the current understanding of the electrical and optical properties of Cu_3–*x*_P is surprisingly poor. Various authors have identified Cu_3–*x*_P as a metal,^14,18,22^ many others as a semiconductor,^16,24,26,28−30,33^ and in one study it was labeled a semimetal.^27^ Device-level applications of Cu_3–*x*_P as a semiconducting photovoltaic absorber^24^ and as a metallic contact^22^ have both been claimed. Furthermore, it is not clear if the electrical properties of Cu_3–*x*_P are modified by changing the composition and, in particular, whether Cu_3–*x*_P can be doped n-type by nonstoichiometry. The single-phase region of Cu_3–*x*_P has only been determined for bulk samples,^13,17,18^ with different results obtained in different studies. Finally, the effect of growth temperature and other parameters on the properties of Cu_3–*x*_P has not been investigated.

Polycrystalline Cu_3–*x*_P thin films are an ideal platform to answer these questions. Unlike the case of nanoparticles, the properties of thin films are not affected by quantum confinement, and electrical and optical properties can be measured more precisely. Compared to single crystals and bulk powders, an advantage of thin films is that their properties can rapidly be characterized as a function of elemental composition and process conditions using high-throughput methods.^34−36^ Previous thin-film work on Cu_3–*x*_P is, however, limited to nonreactive sputter deposition from a Cu_3–*x*_P target,^22^ chemical vapor deposition,^20^ electrodeposition,^19,21^ and phosphorization of metallic Cu^23^ with only basic materials characterization. A deposition technique amenable to high-throughput experiments is reactive RF sputtering in a PH_3_-containing atmosphere. Although this technique has rarely been applied to deposit metal phosphides, we have recently shown its feasibility for various phosphide compounds of current interest.^12,37−39^

In this work, we combine high-throughput experiments on reactively sputtered Cu_3–*x*_P, first-principles calculations, and semiclassical transport theory. The goal is to investigate the electronic nature of Cu_3–*x*_P films as a function of composition and growth conditions. We present strong evidence that Cu_3–*x*_P is a p-type semimetal natively doped by Cu vacancies regardless of overall composition and growth conditions. Its single-phase region is likely limited to a narrow range of Cu-deficient compositions. We find that the density of Cu vacancies increases with deposition temperature. We observe an anisotropic electrical conductivity in excellent agreement with the anisotropy of the hole effective masses. Finally, we identify a near-infrared absorption feature somewhat similar to a localized surface plasmon resonance previously reported in Cu_3–*x*_P nanoparticles. In thin-film samples, this peak is likely to be an intrinsic feature of bulk Cu_3–*x*_P due to interband transitions.

## Experimental and Computational Details

Cu_3–*x*_P thin films were deposited by reactive RF sputtering on borosilicate glass substrates in a PH_3_/Ar atmosphere. For experimental throughput purposes, we deposited compositionally graded films by cosputtering a Cu_3–*x*_P target and a Cu target facing two opposite sides of the substrate. Each data point and spectrum in the article corresponds to one specific point of these combinatorial films.^34−36^

Elemental composition and film thickness were determined by X-ray fluorescence (XRF). X-ray diffraction (XRD) measurements were conducted using Cu Kα radiation and a 2D detector. Sheet resistance was measured in the substrate plane with a collinear four-point probe. Temperature-dependent Hall carrier concentration and mobility were measured in the substrate plane in the van der Pauw configuration. The complex dielectric function and absorption coefficient were extracted by spectroscopic ellipsometry.

First-principles calculations were performed using density functional theory (DFT) within the projector-augmented wave (PAW) formalism^40^ and with a plane-wave basis set as implemented in the GPAW code.^41,42^ The Perdew–Burke–Ernzerhof (PBE) exchange correlation functional^43^ was employed for structural relaxation. The GLLB-SC exchange correlation functional^44^ was employed for electronic ground-state calculations. The complex dielectric function and absorption coefficient were calculated by linear response theory within the Random Phase Approximation (RPA) including local field effects, as implemented in GPAW.^45^ Transport properties of Cu_3–*x*_P as a function of doping density were estimated using Boltzmann transport theory as implemented in BoltzTraP2.^46^ Extensive experimental and computational details are given in the Supporting Information.

## Results

### Growth, Composition, and Structure

#### Growth Routes and Composition

**Figure 1:**
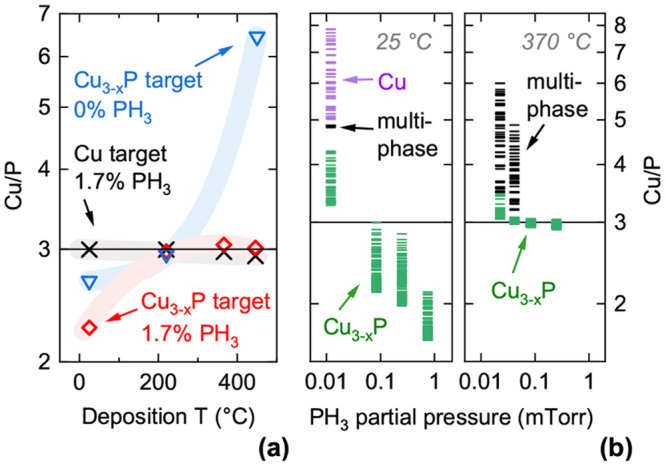
Composition and phase analysis of Cu_3–*x*_P films under different deposition conditions. (a) Film composition versus deposition temperature. Each data set is indicated by a guiding line. (b) Combinatorial film compositions obtained at different PH_3_ partial pressures by cosputtering a Cu and a Cu_3–*x*_P target. Left panel: Room-temperature deposition. Right panel: 370 °C deposition. Data points are colored green, purple, and black when their corresponding XRD patterns contain Cu_3–*x*_P peaks only, Cu peaks only, and both types of peaks, respectively.

**Figure 2:**
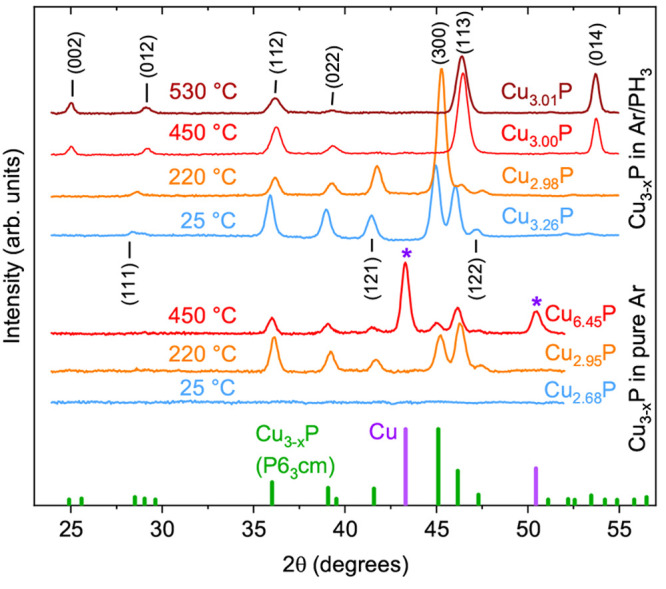
Lower data set: XRD patterns of Cu_3–*x*_P films deposited in pure Ar from a Cu_3–*x*_P target. Upper data set: XRD patterns of Cu_3–*x*_P films deposited in a PH_3_-containing atmosphere by simultaneous sputtering of a Cu_3–*x*_P and a Cu target. Film compositions, deposition temperatures, and reference peaks from powder samples^13^ are indicated. At room temperature, the inclusion of PH_3_ yields polycrystalline (rather than amorphous) films. Including PH_3_ also prevents P losses at high temperatures.

**Figure 3:**
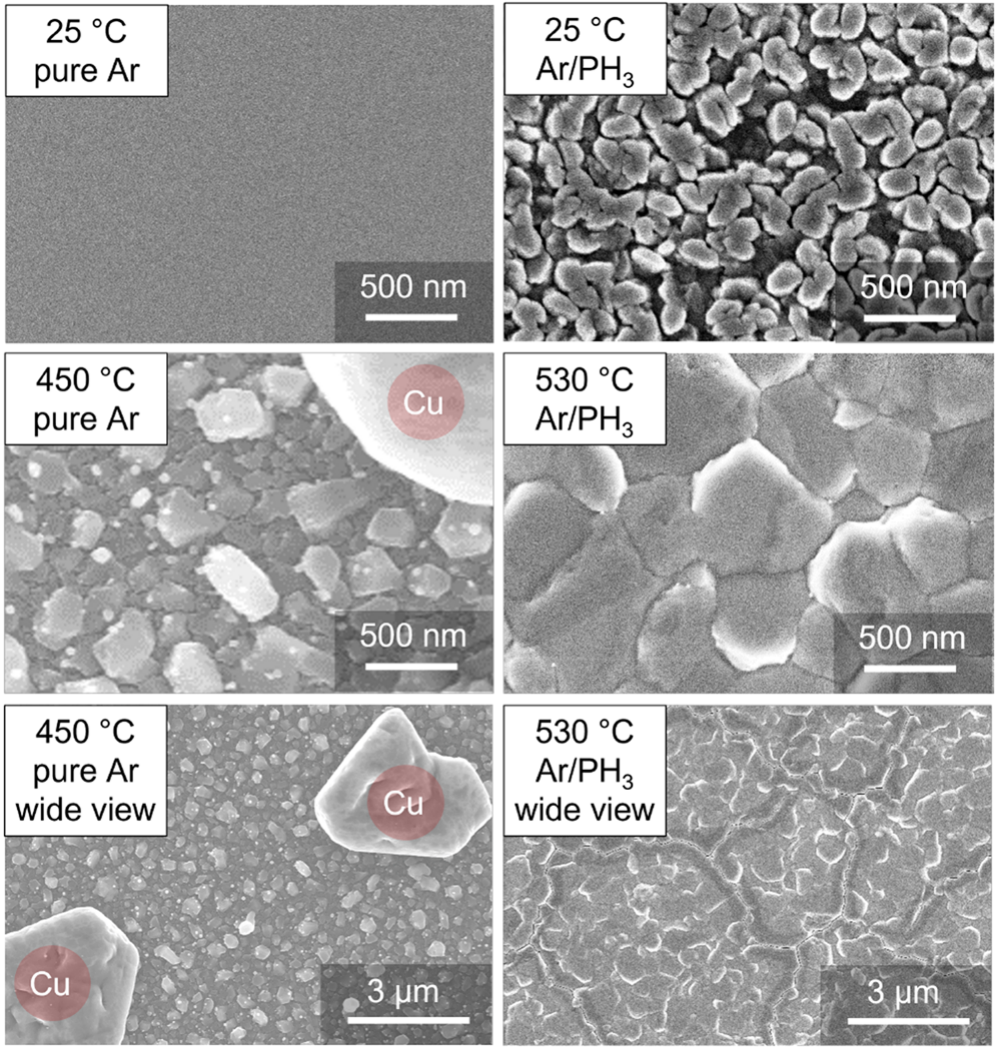
SEM images of Cu_3–*x*_P films deposited in pure Ar from a Cu_3–*x*_P target (left) and in a PH_3_-containing atmosphere from Cu_3–*x*_P and Cu targets (right). A polycrystalline rather than amorphous morphology is found when PH_3_ is included in the growth atmosphere at room temperature (top row). When depositing in pure Ar, most of the phosphorus is already lost at 450 °C, resulting in formation of metallic Cu islands (middle and bottom left). When depositing in Ar/PH_3_, a homogeneous Cu_3–*x*_P film with grains larger than 500 nm can be grown at even higher temperatures (530 °C, middle and bottom right).

We initially deposited Cu_3–*x*_P films by RF sputtering of a Cu_3–*x*_P target in pure Ar, but this process route does not allow the exploration of a broad parameter space. The films rapidly lose P with increasing deposition temperature (Figure 1a), and Cu/P ratios below 3 are only achievable below ∼250 °C. Without intentional heating, films are X-ray amorphous (Figure 2). At 220 °C, polycrystalline Cu_3–*x*_P grows without detectable secondary phases (Figure 2). At 450 °C, polycrystalline Cu_3–*x*_P is still present, but the film has lost most of its P (Cu/P ≃ 6.5) and mainly consists of large Cu islands (Figure 3). The changes in film morphology with deposition temperature are shown in greater detail in Figure S1.

Adding PH_3_ to the growth atmosphere significantly expands the conditions under which polycrystalline Cu_3–*x*_P can be grown. First, the deposition temperature can be increased up to at least 530 °C (Figures 2 and 3). Second, deposition of Cu_3–*x*_P by reactive sputtering of a metallic Cu target becomes possible in the same temperature range as for the Cu_3–*x*_P target (Figure 1a). This is a significant process advantage due to the lower cost, higher purity, and higher sputter rate of a metal target. Finally, polycrystalline (rather than amorphous) Cu_3–*x*_P is obtained when depositing at room temperature (Figures 2 and 3). This unexpected finding is likely due to a plasma-assisted crystallization process driven by the PH_3_ dissociation products^47^ in an RF plasma. In the remainder of the article, we will discuss the properties of Cu_3–*x*_P deposited in a PH_3_-containing atmosphere, unless otherwise specified.

When depositing films at room temperature, a broad range of Cu/P ratios is accessible by tuning the PH_3_ partial pressure (Figure 1b). By simultaneous sputtering of a Cu and a Cu_3–*x*_P target facing two opposite sides of the substrate, combinatorial films with a gradient in the Cu/P ratio can be obtained at a single PH_3_ partial pressure (Figure 1b). When depositing at 370 °C, P-rich compositions with Cu/P ratios significantly less than 3 are no longer accessible (Figure 1b). The lowest Cu/P ratios obtained at 370 °C, 450 °C, and 530 °C are 2.89, 2.87, and 2.91, respectively. Compatibly with our previous study on CuP_2_,^12^ we assume that other Cu–P phases with Cu/P ratios much less than 3 are unstable at these temperatures, due to the high driving force for P evaporation. All Cu_3–*x*_P films were found to be air-stable.

#### Phase Analysis

Regardless of deposition temperature, the XRD patterns of all our Cu_3–*x*_P films close to the stoichiometric point are consistent with the *P*6_3_*cm* structure^13,18^ previously described for bulk samples (see Figure 2). We do not observe any peaks extraneous to the *P*6_3_*cm* structure in any of the films with Cu/P < 3, not even in the extremely P-rich films (up to Cu_1.67_P) deposited at room temperature. Although polycrystalline secondary phases are absent, we note that CuP_2_ and elemental phosphorus tend to form amorphous phases in bulk samples.^17,48^ Even for the case of thin films reactively sputtered at room temperature, we confirmed elsewhere that CuP_2_^12^ and elemental phosphorus^38^ are in amorphous form. Thus, amorphous CuP_2_ and/or P secondary phases are very likely to exist when Cu/P < 2.9, which is the most P-rich composition obtainable at temperatures where CuP_2_ and P are no longer stable in the solid state.

In the Cu-rich region (Cu/P > 3), all films have a composition threshold beyond which XRD peaks from metallic Cu are observed. The range of Cu-rich compositions free of metallic Cu peaks becomes narrower with increasing PH_3_ partial pressure (Figure 1b). As argued in the Supporting Information, this is related to a shift in the prevalent formation mechanism of the Cu_3–*x*_P film, from deposition of Cu_3–*x*_P vapor to incomplete phosphorization of metallic Cu. In general, XRD results indicate that moderately Cu-rich Cu_3–*x*_P films can be grown without precipitation of polycrystalline Cu. However, SEM characterization reveals that some secondary phases are already present at Cu_3.00_P composition in films deposited above 370 °C (Figure S2). These phases are likely to be noncrystalline Cu. They are no longer visible by SEM at Cu_2.95_P composition (Figure S2). Thus, the single-phase region of Cu_3–*x*_P films deposited at 370 °C and above is probably not wider than the Cu_2.9_P–Cu_3.0_P range, based on our SEM results. Films deposited at room temperature might have an extended single-phase region on the Cu-rich side, since we could not clearly distinguish secondary phases by SEM in these films. In general, formation of amorphous CuP_2_ (at low temperatures) or P loss (at high temperatures) constrains the single-phase region on the P-rich side. Formation of a Cu secondary phase constrains the single-phase region on the Cu-rich side.

In qualitative agreement with our results, previous studies of bulk Cu_3–*x*_P found that single-phase samples always had Cu/P < 3,^13,17,18,49^ although different single-phase stability ranges were found in each study. The stability of these P-rich compositions was generally attributed to a high concentration of Cu vacancies (V_Cu_), responsible for the high p-type conductivity in Cu_3–*x*_P. Previous first-principles calculations confirmed that one V_Cu_ per 24-atom unit cell should be thermodynamically stable under a wide range of chemical potentials.^26^ In the following sections, we will assume that V_Cu_ defects generate most of the charge carriers in Cu_3–*x*_P films. This assumption will, however, be justified throughout the article based on our own data.

#### Structural Properties

**Figure 4:**
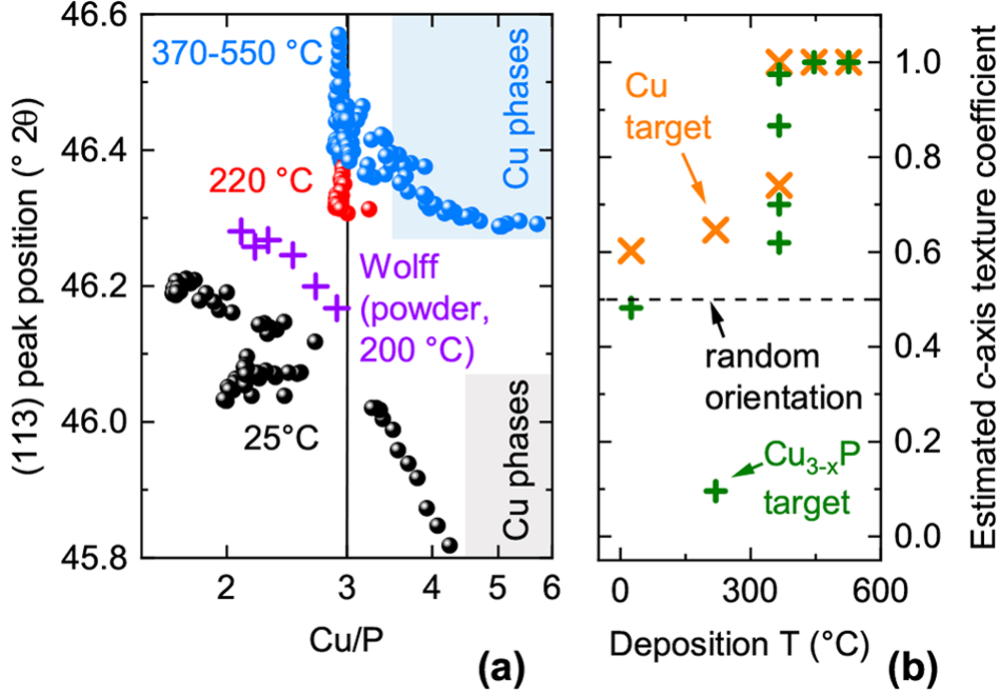
Structural parameters of Cu_3–*x*_P films. (a) Position of the (113) XRD peak as a function of film composition, grouped by deposition temperature. Analogous data from Cu_3–*x*_P powders grown at 200 °C by Wolff et al.^18^ are shown. The composition regions where metallic Cu peaks are observed in XRD are shaded in gray (room-temperature deposition) and in blue (370 °C deposition), following the data in Figure 1b. (b) Estimated *c*-axis texture coefficient versus deposition temperature, grouped by the target used for deposition. A coefficient of one indicates that the *c*-axis is perpendicular to the substrate plane. A coefficient of zero indicates that the *c*-axis lies in the substrate plane. The method used to estimate the texture coefficient is described in the Supporting Information.

To evaluate structural changes in Cu_3–*x*_P as a function of composition and deposition temperature, we analyze the position of the (113) XRD peak (Figure 4a). In general, the (113) peak shifts to higher diffraction angles (shorter interplanar distances) with increasing deposition temperature and with decreasing Cu/P ratio. The other peaks in the XRD patterns tend to shift in the same direction (see Figure 2 for some examples). The relative magnitude of the shifts in each XRD pattern is generally compatible with a multiplication of the three lattice constants by a common factor. Hence, we conclude that the (113) peak shifts are indicative of lattice contraction or expansion in all three dimensions. Our data confirm and extend the results by Wolff et al.,^18^ who reported contraction of the unit cell in all directions in Cu_3–*x*_P powders with decreasing Cu/P ratios (Figure 4a).

For films deposited at 370 °C, the (113) peak stops shifting at high Cu/P ratios, roughly corresponding to the region where we observe precipitation of polycrystalline Cu (blue shaded area in Figure 4a). This suggests that all excess Cu precipitates as a secondary phase rather than being incorporated in the Cu_3–*x*_P lattice. For films deposited at room temperature, the (113) peak shift is generally more pronounced up to the precipitation threshold for polycrystalline Cu. Hence, low-temperature-grown Cu_3–*x*_P may be able to incorporate substantial Cu excess in the form of defects, a phenomenon that may be linked to the lower volatility of P at low temperatures. This would be a qualitative difference with Cu_3–*x*_P grown at higher temperatures, where evidence for Cu precipitation already at Cu/P = 3 has been shown in bulk samples^13,17,18,49^ and thin-film samples (this work). As will be shown later, changes in lattice constants are generally correlated to changes in the concentration of Cu vacancies in the different films.

The texture coefficient of Cu_3–*x*_P films is shown in Figure 4b as a function of deposition temperature. The *c*-axis of the Cu_3–*x*_P crystallites has an increasing tendency to align perpendicular to the substrate plane with increasing temperature. Similar *c*-axis texturing effects are often observed in other uniaxially anisotropic materials.^50−52^ We also observe that films grown from the Cu target are generally more *c*-axis textured than films grown from the Cu_3–*x*_P target at the same temperature (Figure 4b and Figure S3).

Finally, Raman spectra of Cu_3–*x*_P films do not exhibit any peaks (Figure S4), even though various Raman-active modes are expected for space group *P*6_3_*cm*.^53^ Raman peaks compatible with a few previous studies^32,33^ can only be observed at very high laser excitation intensities above the ablation threshold of Cu_3–*x*_P, raising questions on the validity of these previously reported spectra.

### Electrical Properties

#### Composition Dependence

**Figure 5:**
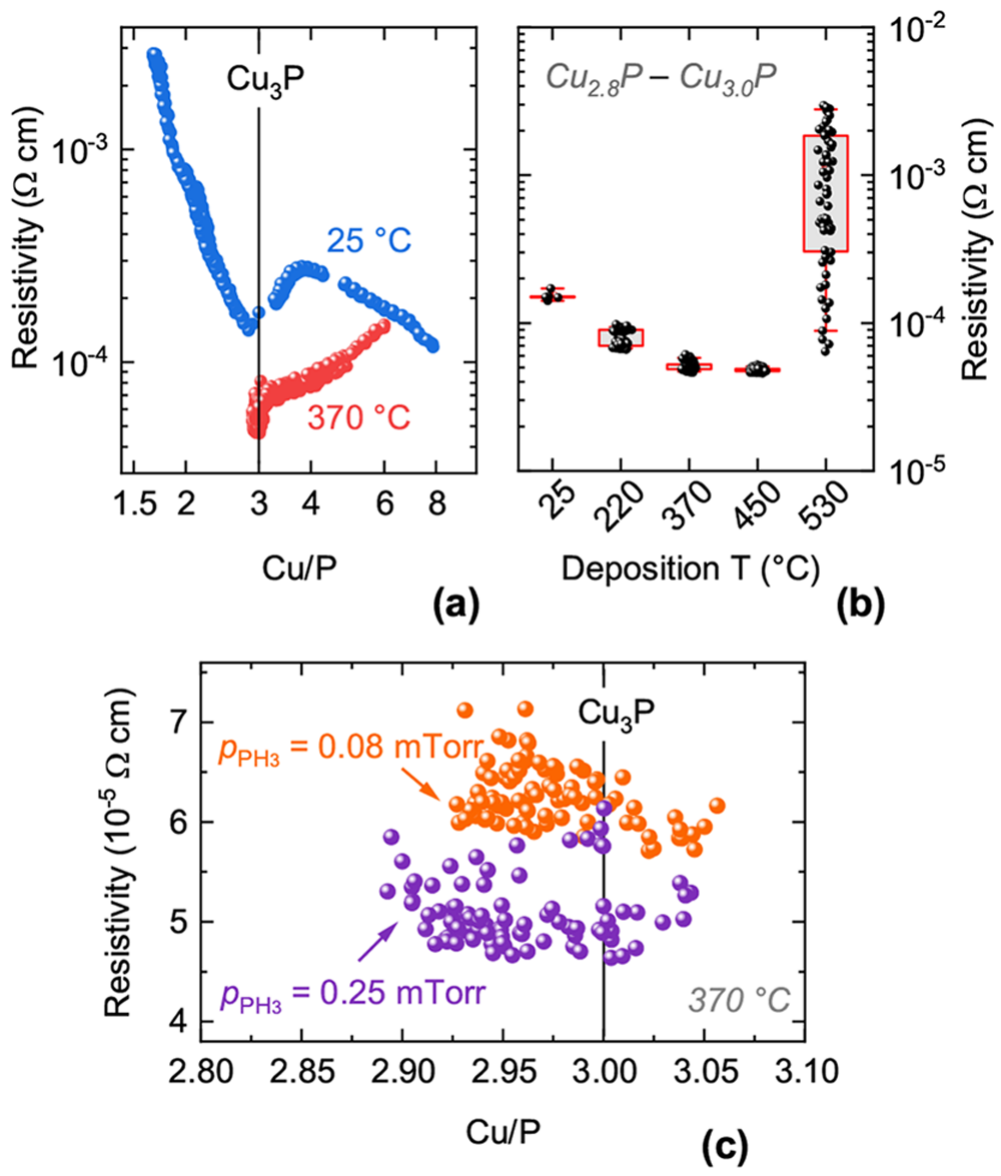
Resistivity of Cu_3–*x*_P films. (a) Resistivity versus composition for two different deposition temperatures. (b) Resistivity versus deposition temperature (with boxplots) for films in the 2.8 < Cu/P < 3.0 composition range. (c) Resistivity near the Cu_3_P stoichiometric point as a function of PH_3_ partial pressure at 370 °C deposition temperature. The data comes from two combinatorial depositions with different PH_3_ partial pressures. Other material properties (composition ranges, thicknesses, texture coefficients) are very similar in the two data sets.

The resistivity of the Cu–P system can be mapped over a broad region, from Cu_1.7_P to Cu_8.0_P, in films deposited at room temperature (Figure 5a). A local resistivity minimum (1.4 × 10^–4^ Ω cm) exists at Cu/P ≃ 2.83. The position of the minimum is close to the most P-rich composition that could be obtained at temperatures above 370 °C (Cu_2.87_P). This result indicates that further electrical doping by nonstoichiometry is not possible for Cu/P ratios lower than this threshold. Thus, P excess is accommodated by secondary phases rather than defects, in line with the arguments in the previous section. The steep increase in resistivity on the left side of the minimum is due to the much higher resistivity of CuP_2_ (around 1 Ω cm) with respect to Cu_3–*x*_P, as shown elsewhere.^12^

On the right side of the minimum, the resistivity increases until the Cu_3.8_P composition is reached (Figure 5a). Since metallic Cu peaks begin to appear in XRD at a similar composition (Figure 1b), we assume that the resistivity increase is caused by changes in concentration of point defects up to the Cu precipitation threshold. These defects are also likely to be the cause of unit cell expansion up to a composition of roughly Cu_4.5_P (Figure 4a). Beyond this threshold, a parallel transport channel along highly conductive Cu percolation paths causes the resistivity to decrease again.

**Figure 6:**
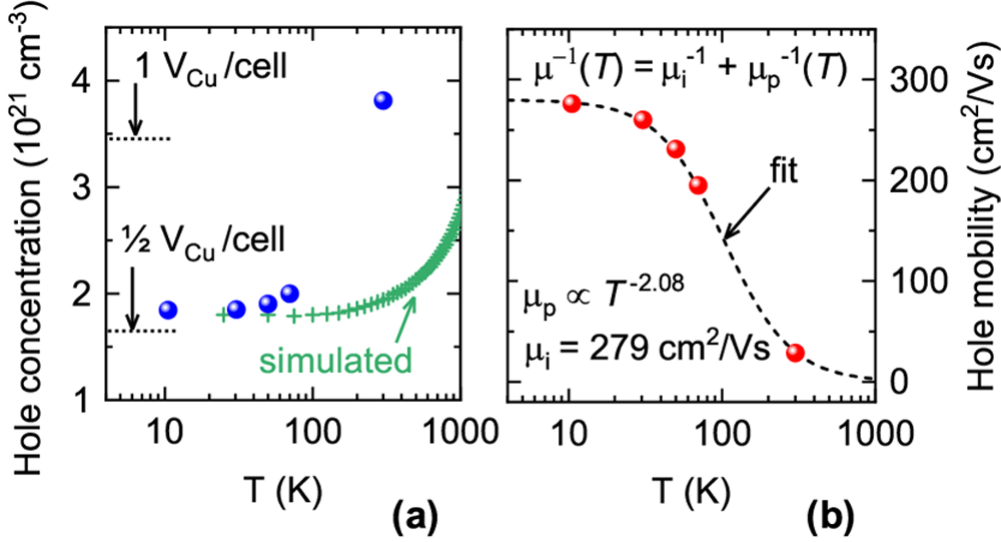
Temperature-dependent Hall carrier concentration (a) and Hall mobility (b) of a Cu_2.95_P film deposited at 370 °C. The hole concentration expected from half and one uncompensated Cu vacancy per 24-atom unit cell (single acceptor) is indicated in (a). Also shown in (a) is the simulated temperature dependence of the Hall hole concentration, as calculated by Boltzmann transport theory on Cu_3_P with a Fermi level 0.3 eV below its intrinsic value. The temperature-dependent mobility in (b) is fitted considering two parallel scattering channels (ionized impurities and optical phonons) resulting in the fitting equation shown in the upper part of (b). The best-fit parameters are shown in the lower part of (b).

The composition dependence of the resistivity in films deposited at 370 °C (Figure 5a) has some qualitative differences. The minimum resistivity is still found in the vicinity of the stoichiometric point (4.6 × 10^–5^ Ω cm). This value is very similar to the resistivity measured in Cu_3–*x*_P single crystals (5 × 10^–5^ Ω cm)^14^ and a factor of 2 lower than in sintered powders (about 1.0 × 10^–4^ Ω cm) .^15,18^ Hall measurements on a Cu_2.95_P film indicate p-type conductivity with hole concentration of 3.81 × 10^21^ cm^–3^ and hole mobility of 28.8 cm^2^/(V s) at room temperature (Figure 6). P-type conductivity is confirmed by measurement of a Seebeck coefficient of +11.2 μV/K in an analogously deposited film (Figure S5).

The Cu/P < 2.9 region is not experimentally available at a 370 °C deposition temperature, as explained in the previous section. When Cu/P > 3.0, the resistivity continues to increase up to the boundary of the investigated composition range (Cu/P ≃ 6), despite the fact that Cu secondary phases are already observed at much lower Cu/P ratios (Figure 1b). This indicates that transport along Cu_3_P channels is a lower-resistance path in these films, even under a substantial presence of Cu secondary phases.

Interestingly, the resistivity in the 3.2 < Cu/P < 3.6 range follows the same trend in two distinct sets of samples: One in which metallic Cu peaks are present in XRD, and another in which they are absent (Figure S2). This finding suggests that a certain amount of Cu secondary phases exist even when they are not detected by XRD, confirming the SEM observations in Figure S2. Thus, formation of metallic Cu appears to be favored over formation of compensating donors in Cu-rich films. The moderate increase in (113) interplanar spacing for Cu/P > 3 (Figure 4a) may simply be due to a decrease in V_Cu_ concentration. As a consequence, the p-type doping effect of Cu vacancies is never fully compensated by native donors, and Cu_3–*x*_P films are always p-type, even when Cu/P > 3.0. Accordingly, we did not measure any negative Seebeck coefficients in any film in the 2.9 < Cu/P < 3.6 range.

The average resistivity of films in the vicinity of the stoichiometric point (2.8 < Cu/P < 3.0) decreases with increasing deposition temperature up to 450 °C (Figure 5b). At a higher temperature (530 °C), a higher average resistivity and a much higher standard deviation are observed. This phenomenon is probably linked to an extrinsic effect, i.e., the microscopic cracks visible in SEM images (Figure 3), which are likely detrimental for electrical transport. Analysis of the optical properties (shown later) indicates that the general decrease in resistivity with increasing temperature is likely due to an increase in hole concentration rather than in hole mobility.

A detailed view of the composition-dependent resistivity data close to the stoichiometric point (Figure 5c) reveals that the resistivity depends on the PH_3_ partial pressure during deposition but not on film composition. The overall decrease in resistivity with increasing PH_3_ partial pressure (20% lower resistivity with 3 times higher partial pressure) is expected, since a higher P chemical potential relative to the Cu chemical potential leads to a lower formation energy for Cu vacancies.^26^ The complete lack of correlation between resistivity and film composition is more difficult to rationalize, since a higher concentration of Cu vacancies (and thus a lower resistivity) should result in more P-rich compositions. Possible explanations are provided in the Discussion section.

#### Temperature Dependence

Hall effect measurements show that the hole concentration of a low-resistivity Cu_3–*x*_P film decreases by a factor 2 from 300 K to 10 K (Figure 6a), in quantitative agreement with previous measurements on powder samples.^18^ On the other hand, the hole mobility increases with decreasing temperature up to 276 cm^2^/(V s) at 10 K, indicating that phonon scattering is the main mobility-limiting mechanism above a few tens of kelvin. At lower temperatures, we expect ionized impurity scattering to be responsible for flattening of the mobility, due to the high density of acceptor defects in Cu_3–*x*_P.

We fit the experimental hole mobility μ(*T*) with the expression μ^–1^(*T*) = μ_i_^–1^ + μ_p_^–1^(*T*). Here, μ_i_ is the mobility resulting from the ionized impurity scattering channel and μ_p_(*T*) is the mobility resulting from the phonon scattering channel. We assume that ionized impurity scattering is roughly temperature-independent in a highly doped material like Cu_3–*x*_P^39,54^ and that phonon-limited mobility can be described by a power law.^55^ The fit yields μ_i_ = 279 cm^2^/(V s) and μ_p_ = *aT*^–2.08^, where *a* is a temperature-independent factor (Figure 6b). The low-temperature mobility of this Cu_3–*x*_P film is remarkably high for a nonepitaxial polycrystalline thin-film material with such a high carrier concentration. An explicit comparison with other materials is provided in the Supporting Information. The mobility of the present Cu_3–*x*_P film is also much higher than the mobility of sintered Cu_3–*x*_P powders presented in a previous study (7.4 cm^2^/(V s) at 300 K and 38 cm^2^/(V s) at 2 K).^18^ Thus, we conclude that Cu_3–*x*_P films have very low ionized impurity scattering rates, despite the high density of such impurities (V_Cu_ acceptors). The ionized impurity scattering rate in a highly doped material is expected to scale with , where m* is the carrier effective mass and ε_*s*_ is the static dielectric constant.^56^ Since the calculated m* for holes in Cu_3–*x*_P at the observed carrier concentration is not unusually low (see Discussion section), it is likely that Cu_3_P has a high static dielectric constant. In fact, high values of ε_*s*_ are often encountered in narrow band gap semiconductors and semimetals.^57^ We emphasize that the electrical properties extracted from Hall measurements are derived under the assumption of a single carrier type, i.e., with holes being much more abundant than electrons. The next sections show that this assumption is probably justified for Cu_3–*x*_P.

#### Metal, Semimetal, or Degenerately Doped Semiconductor?

The electrical properties of the present Cu_3–*x*_P films could be compatible with a metal or a degenerately doped semiconductor. However, previous studies do not agree on to which of the two classes Cu_3–*x*_P belongs. Some authors identified it as a metal,^14,18,22^ others as a semiconductor.^16,24,26,28−30,33^

Density functional theory (DFT) calculations with the PBE exchange correlation functional^43^ yield metallic band structures, both for stoichiometric Cu_3_P^58^ and for Cu_3–*x*_P with 1 V_Cu_/unit cell.^26^ This is not conclusive evidence that Cu_3–*x*_P is a metal, because semiconducting compounds are often incorrectly predicted to be metals at the PBE level due to the well-known “band gap problem” of semilocal functionals.^59^ However, stoichiometric Cu_3_P is also predicted to be a metal using the hybrid HSE06 functional,^60^ with which band gaps are not systematically underestimated.^61^ This substantiates the hypothesis that Cu_3–*x*_P is intrinsically a metal rather than a degenerately doped semiconductor.

**Figure 7:**
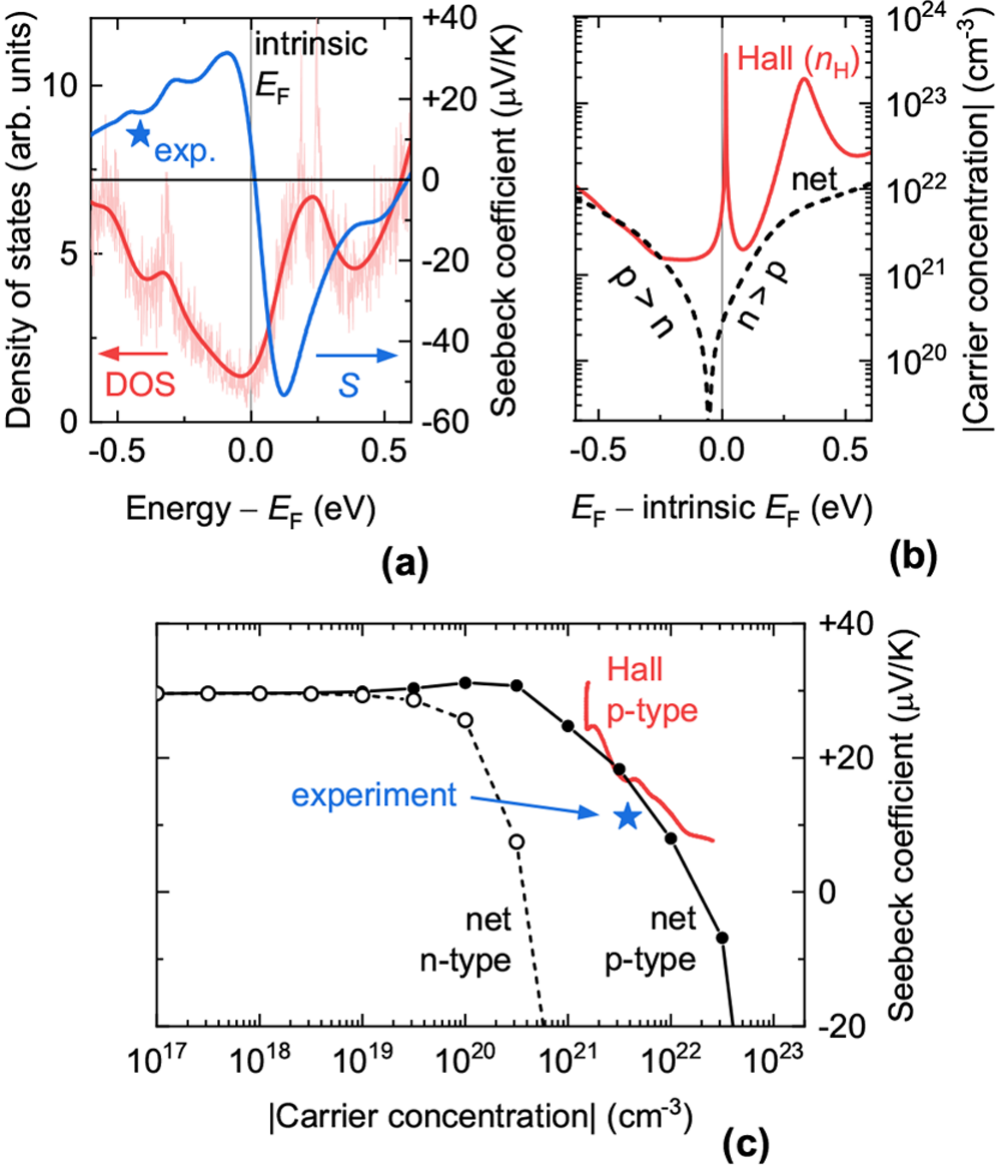
Simulated electronic and transport properties of Cu_3_P at various doping levels. (a) Total electronic DOS of pristine Cu_3_P by DFT (PBE level), extracted from the Materials Project database.^58^ The calculated DOS with and without thermal broadening is shown. The low—but not zero—DOS at the Fermi level indicates semimetallicity. Also shown is the simulated Seebeck coefficient as a function of Fermi level position with respect to the intrinsic Fermi level of pristine Cu_3_P (zero of the energy scale). The experimental Seebeck coefficient of a Cu_3–*x*_P film (star marker) is shown for comparison. (b) Simulated absolute value of the net carrier concentration *p* – *n* and of the Hall carrier concentration *n*_*H*_ as a function of Fermi level position with respect to the intrinsic Fermi level of pristine Cu_3_P (zero of the energy scale). (c) Simulated Pisarenko plots based on the data in (a) and (b). The black data are plotted with the net carrier concentration as the abscissa (p-type for solid data points, n-type for hollow data points). The red data are plotted with the Hall carrier concentration as the abscissa. The star marker indicates the experimental Seebeck coefficient versus experimental Hall hole concentration.

The total density of states (DOS) calculated for stoichiometric Cu_3_P at the PBE level (Materials Project ID: mp-7463)^58^ is shown in Figure 7a. The DOS calculated at the HSE level is similar.^60^ The DOS at the Fermi level is low, with a minimum in its immediate vicinity. Metallic materials with these features (such as graphite, As, and Bi) are often referred to as semimetals, to indicate that there is a small overlap between hole-like bands and electron-like bands at the Fermi level. The limited availability of states at the Fermi level results in smaller charge carrier concentrations than in conventional metals.^62^ In addition, semimetals may shift between n-type and p-type behavior with temperature or under relatively small changes in the Fermi level driven by perturbations such as doping, strain, or biasing.^63^

#### Semiclassical Simulation of Transport Properties

It is desirable to check if the experimentally determined properties of Cu_3–*x*_P films are compatible with a semimetal. For a direct comparison, we simulated transport properties of Cu_3_P at 300 K from the calculated band structure of Cu_3_P at the PBE level (Materials Project ID: mp-7463).^58^ Net carrier concentrations were derived from the DFT-computed density of states (Figure 7a) and the Fermi distribution function. Other transport quantities (electrical conductivity, conductivity effective masses, Hall carrier concentration, Seebeck coefficient) were calculated by semiclassical Boltzmann transport theory based on an interpolation of the DFT-calculated band structure.^46,64^ Two important approximations of the present implementation of Boltzmann transport theory are (i) the carrier scattering time is constant across all bands and energies (we chose 10 fs for reasons explained in the Supporting Information); (ii) doping affects the Fermi level position, but not the band structure itself. The net carrier concentration is defined as |*p* – *n*|, where *p* is the free hole concentration and *n* is the free electron concentration (both are positive quantities). These carriers can be either intrinsic to the material due to a nonzero DOS at the Fermi level or can be generated by doping.

The Fermi level of intrinsic, defect-free Cu_3_P is predicted to lie very close to the DOS minimum (Figure 7a). This intrinsic Fermi level is indicated as the zero of the energy scale in Figure 7a,b. The net carrier concentration in this hypothetical Cu_3_P without any dopants is 3 × 10^20^ cm^–3^ (about one net electron per 250 atoms; see dashed line in Figure 7b). This intrinsic electron concentration arises from the nonzero DOS (and, thus, nonzero free electron density) of Cu_3_P at the Fermi level. As the Fermi level shifts down through p-type doping, the net carrier concentration vanishes around 60 meV below the intrinsic Fermi level. At this energy, the intrinsic electrons are fully compensated by holes generated by doping. Upon further p-type doping, holes dominate the conductivity rather than electrons. This net carrier concentration is not directly measured by the Hall effect. Instead, the Hall carrier concentration (positive or negative) is defined as *n*_*H*_ = 1/*eR*_*H*_, where *R*_*H*_ is the Hall coefficient and *e* is the elementary charge. When both electrons and holes contribute to the conductivity
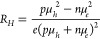
1where μ_*h*_ and μ_*e*_ are the (positive) mobilities of holes and electrons, respectively.^65^ In the transport calculations, the mobilities are obtained from the conductivity effective masses *m*_*e*,h_^*^(46,64) and the assumed scattering time τ as μ_*h*,*e*_ = *eτ*/*m*_*e*,h_^*^. From eq 1, it is apparent that the Hall concentration deviates from the simple net carrier concentration (*p* – *n*), unless electrons and holes have very different concentrations. This discrepancy is highlighted by calculating (*p* – *n*) and *n*_*H*_ in Cu_3_P as a function of the Fermi level (Figure 7b). The discrepancy between (*p* – *n*) and *n*_*H*_ is particularly evident in materials predicted to be semimetallic (like Cu_3_P) because of the comparable concentrations of electrons and holes. Some counterintuitive features arise from this effect: (1) the Hall carrier concentration is predicted to always be above 1.5 × 10^21^ cm^–3^ even when the net carrier concentration is very low; (2) the Hall carrier concentration is expected to diverge about 10 meV above the intrinsic Fermi level; and (3) there is a small Fermi level range where the simulations predict that Cu_3_P will appear as p-type from a Hall measurement, even though electrons are more abundant than holes. These points are further discussed in the Supporting Information.

The calculated Seebeck coefficient *S* changes sign at roughly the same Fermi level as *R*_*H*_ (Figure 7a). When both electrons and holes contribute to the conductivity,
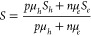
2where *S*_*h*_ (positive) and *S*_*e*_ (negative) are the Seebeck coefficients for holes and electrons alone, respectively.^65^ As expected, lowering and raising the Fermi level in the simulation leads to positive and negative Seebeck coefficients, respectively. In summary, a Hall effect measurement and a thermovoltage measurement on a Cu_3–*x*_P sample are expected to yield the same conductivity type.

#### Comparison with Experiment

The experimentally measured Hall carrier concentration at 300 K (3.81 × 10^21^ cm^–3^) corresponds to a Fermi level 0.41 eV below the intrinsic Fermi level according to Figure 7b. The experimental and calculated Seebeck coefficients at this Fermi level (+11.2 μV/K and +16.6 μV/K, respectively) are in reasonable agreement. This is also visualized in the Pisarenko plot (Seebeck coefficient versus carrier concentration) in Figure 7c.

**Figure 8:**
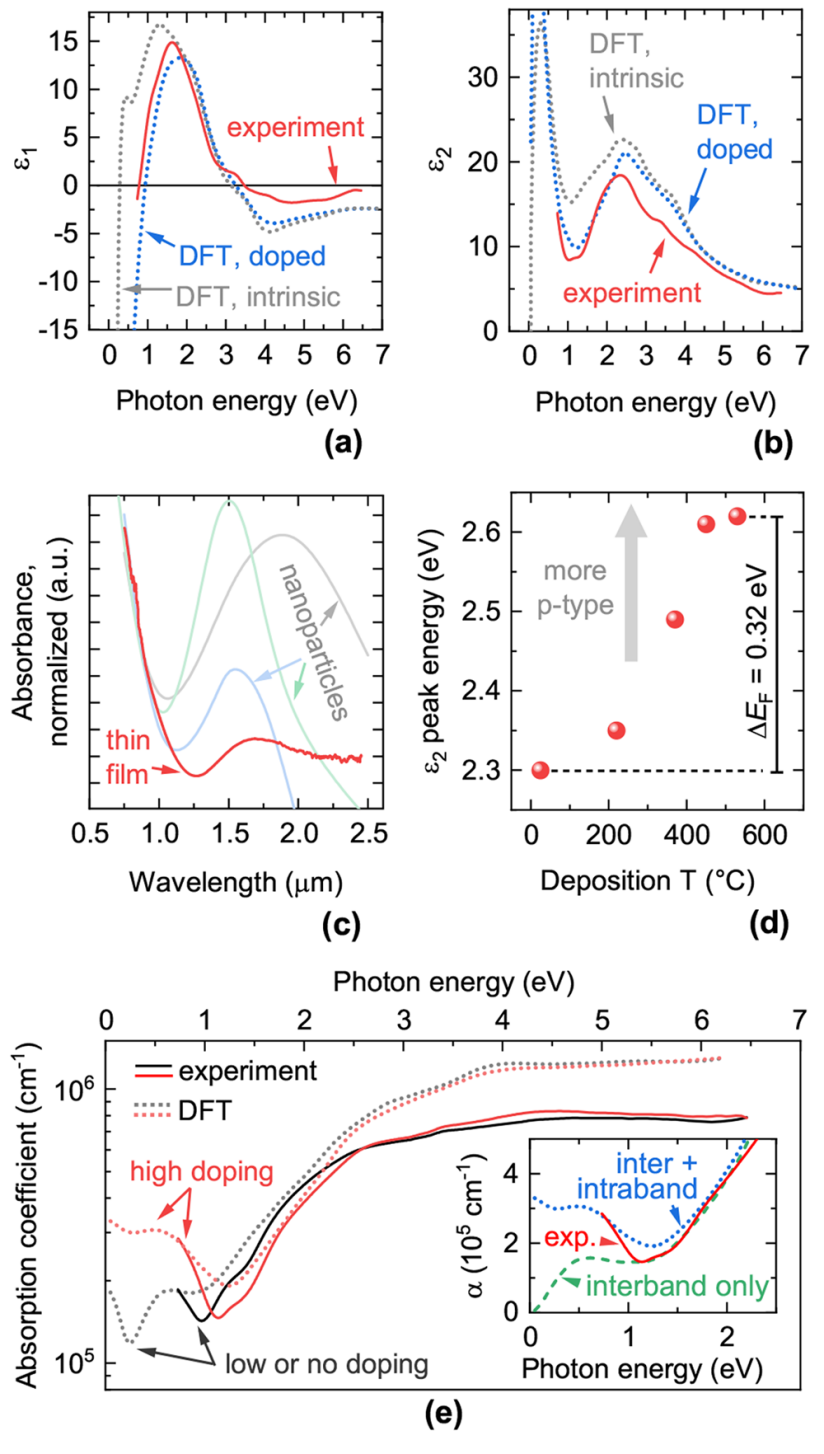
Optical properties of Cu_3–*x*_P films. (a, b) Real (ε_1_) and imaginary part (ε_2_) of the dielectric function. Experimental spectra were measured by ellipsometry. Simulated spectra were calculated by DFT on intrinsic Cu_3_P and on doped Cu_2.83_P with 1 V_Cu_/unit cell. (c) NIR absorbance derived from transmission and reflection measurements. Previously reported absorbances of Cu_3–*x*_P nanoparticles^24,26,29^ are also shown. (d) Photon energy of the peak found in experimental ε_2_ spectra (see Figure S6) as a function of deposition temperature. The shift in peak energy is attributed to a Burstein–Moss shift. (e) Absorption coefficient α. The experimental α from a high-resistivity Cu_3–*x*_P film is compared to the DFT-calculated α for intrinsic Cu_3_P (“low or no doping” label). The experimental α from a low-resistivity Cu_3–*x*_P film is compared to the DFT-calculated α for doped Cu_2.83_P (“high doping” label). (e, inset) Comparison of experimental and calculated α spectra in the IR. One calculated spectrum (dashed line) only includes interband transitions. The other calculated spectrum (dotted line) includes both intraband and interband transitions.

Our experimental results are consistent with a p-type semimetal doped with approximately one Cu vacancy per 24-atom unit cell, for two reasons. First, we expect the Fermi level of Cu_3–*x*_P with 1 V_Cu_/unit cell to lie about 0.3 eV lower than the Fermi level of a pristine Cu_3_P semimetal. This Fermi level position is estimated by aligning the localized Cu 3d states of the respective band structures,^26^ following a standard methodology used in band alignment measurements by photoemission spectroscopy.^66^ The magnitude of the Fermi level shift is experimentally confirmed by quantifying the blue shift of the absorption coefficient spectrum of doped Cu_3–*x*_P versus pristine Cu_3_P (Figure 8e, discussed later).

Second, the hole concentration corresponding to one ionized Cu vacancy per unit cell (3.3 × 10^21^ cm^–3^) minus the intrinsic free electrons concentration (3 × 10^20^ cm^–3^) is 3.0 × 10^21^ cm^–3^. This value is close to the experimentally determined Hall carrier concentration of 3.8 × 10^21^ cm^–3^ at 300 K (Figure 6a). At these p-type doping levels, *p* ≫ *n* and the Hall carrier concentration closely follows the net carrier concentration (Figure 7b). Hence, interpreting Hall effect data assuming a single carrier type is justified, and the Hall mobility (Figure 6b) should reflect the mobility of the holes. The experimentally observed decrease in Hall hole concentration with decreasing temperature is confirmed by Boltzmann transport theory, although not quantitatively. A more detailed discussion is given in the Supporting Information. To conclude, the electrical properties of Cu_3–*x*_P films are generally compatible with a semimetallic band structure.

### Optical Properties

#### Optical Signatures of Semimetallicity

The optical properties of Cu_3–*x*_P films provide additional evidence that Cu_3–*x*_P is a semimetal rather than a semiconductor. The real and imaginary parts of the dielectric function (ε_1_ and ε_2_, respectively) were measured by ellipsometry on low-resistivity films (Figure 8a,b). When the photon energy decreases from the visible to the infrared (IR), ε_1_ becomes negative and ε_2_ increases, as expected for a material with a high concentration of free carriers.^67^

We also calculated the dielectric function by DFT using linear response theory and the random phase approximation^41^ (details in the Supporting Information). The calculation on intrinsic Cu_3_P is in reasonably good agreement with the experimental spectra (Figure 8a,b). However, the sign change of ε_1_ in the IR occurs at ∼0.5 eV lower photon energy than in the experimental spectrum. This may be due to the higher carrier concentration in the experimental sample (∼4 × 10^21^ cm^–3^) than in the intrinsic Cu_3_P system considered in the calculation (∼3 × 10^20^ cm^–3^; see previous section). To test this hypothesis, we repeated the dielectric function calculation on structurally relaxed Cu_2.83_P with one Cu vacancy per unit cell. Indeed, the sign change of ε_1_ shifts to higher photon energies, and the agreement with experiment becomes very good over the whole 0.7 eV–6.5 eV spectral range (Figure 8a,b). Again, we emphasize that the calculated dielectric function was derived from an originally semimetallic band structure under the presence of one Cu vacancy per unit cell. Thus, the agreement between experiment and theory is a further indication that Cu_3–*x*_P films are p-type semimetals doped by about one Cu vacancy per unit cell.

#### Near-Infrared Response

To obtain more experimental information on the optical properties of Cu_3–*x*_P films in the near-infrared (NIR), we determined their absorbance from transmission and reflection measurements with an extended IR range (Figure 8c). Interestingly, the films exhibit a NIR absorption peak rather than the continuously increasing absorbance with decreasing photon energy characteristic of free carrier absorption. The peak maximum is around 1.7 μm (0.73 eV). As shown in Figure 8c, the peak position and width are compatible with previous reports of a localized surface plasmon resonance (LSPR) peak in Cu_3–*x*_P nanoparticles,^24,26,68^ although its intensity relative to the main absorption band in the visible is lower than that of the LSPR peak.

Since our sample is a continuous film rather than disconnected nanoparticles, the NIR peak might be due to a (nonlocalized) surface plasmon polariton (SPP) instead of a LSPR. Surface plasmons cannot be excited by light at perfectly planar surfaces due to the requirement of momentum conservation between the plasmon and the photon.^69^ However, the presence of substantial surface roughness can relax this requirement, so SPPs have been observed, for example, in rough Ag films by simple reflection measurements.^69,70^

A problem with the interpretation of the NIR peak as an SPP is that this peak is observed in all our measured Cu_3–*x*_P samples—even in thinner films processed at room temperature, which have very low surface roughness. An alternative interpretation of the NIR peak is offered by plotting DFT-calculated absorption coefficients (Figure 8e), which do not include any plasmonic effects and are only representative of the bulk optical properties of Cu_3–*x*_P. Intriguingly, the calculations for both intrinsic Cu_3_P and p-type doped Cu_2.83_P reveal a peak centered at around 0.6 eV photon energy. When only interband transitions are included in the calculation on doped Cu_2.83_P (inset of Figure 8e, green dashed line), the NIR peak is still present but the absorption coefficient approaches zero at zero photon energy. When both interband and intraband transitions are included in the calculation (inset of Figure 8e, blue dotted line), the NIR peak exists on top of an additional background of increasing absorption coefficient with decreasing photon energy. This background is due to free carrier absorption, i.e., the absorption of low-energy photons by carriers near the Fermi level, which are promoted to states close in energy available within the same band. Note that this free carrier absorption background occurs at much lower photon energies in intrinsic Cu_3_P (up to 0.3 eV) than in doped Cu_2.83_P (up to 1.2 eV) as expected from Drude theory.

To sum up, the NIR peak in Cu_3–*x*_P is likely due to bulk interband transitions, which are independent of both plasmonic effects and the free carrier density. A simultaneous SPP response in the same spectral range cannot be excluded, but it is difficult to deconvolve, due to overlap with the bulk response. The findings presented in this section can help rationalize why the NIR peak in thin films is much less intense than in nanoparticles and why the absorbance sharply decreases again at longer wavelengths in nanoparticles but not in films (Figure 8c). The first effect occurs because the NIR peak has a different origin in the two cases (bulk interband transitions in the films, LSPR in the nanoparticles). The second effect occurs because intraband transitions in very small nanoparticles are obscured by their intense surface plasmonic response. This does not occur in a thicker film free of quantum confinement effects.

#### Burstein–Moss Shift

Comparing again the measured absorption coefficients of the two Cu_3–*x*_P films shown in Figure 8e, we notice that the higher-doping (lower-resistivity) film exhibits an overall blue-shift of the absorption coefficient spectrum at all measured photon energies (Figure 8e). This effect is not related to absorption by free carriers or plasmons. Instead, it has a similar origin as the Burstein–Moss effect observed in degenerately doped semiconductors.^71,72^ It is caused by the lowering of the Fermi level with increasing p-type doping. The lower Fermi level causes optical transitions to collectively shift to higher photon energies, because the initial states of the transitions are shifted to deeper energies in the Cu_3–*x*_P band structure, so higher-energy transitions are required to reach the same final states. Similar to other optical features of Cu_3–*x*_P, the Burstein–Moss shift is also predicted by DFT, as evident by comparing the measured and calculated absorption coefficients in Figure 8e.

We quantified the Burstein–Moss shifts in the lowest-resistivity films at each deposition temperature by finding the photon energy at which their ε_2_ has a maximum (Figure S6). The Burstein–Moss shift become progressively larger as the temperature increases (Figure 8d), indicating that films deposited at higher temperatures are more p-type. This result shows that the electrically probed decrease in resistivity with deposition temperature (Figure 5b) is at least partially due to an increase in hole concentration. It also shows that the increase in resistivity at 530 °C deposition temperature (Figure 5b) is due to a decrease in mobility, probably due to cracks in the films. If it were due to a decrease in hole concentration, the Burstein–Moss shift would not keep increasing at this temperature. The continuously increasing hole concentration with deposition temperature follows the previously predicted increase in Cu vacancy concentration with temperature beyond 1 V_Cu_/unit cell using Boltzmann statistics.^26^

Finally, we note that the difference in the Burstein–Moss shifts between the films with the smallest and largest shifts (deposited at room temperature and 530 °C, respectively) is 0.32 eV. This result is compatible with our calculations of carrier concentration versus Fermi level, which predicted a lowering of the Fermi level by 0.415 eV between intrinsic Cu_3_P and Cu_3–*x*_P with *n*_*H*_ = 3.8 × 10^21^ cm^–3^ (Figure 7b). It is also compatible with the ∼0.3 eV Fermi level down-shift extracted from the previously calculated band structure of Cu_2.83_P with a 1 V_Cu_/unit cell.^26^ These findings confirm that experimental Cu_3–*x*_P samples are semimetals rather than conventional metals, because the much higher DOS at the Fermi level characteristic of a conventional metal would imply a much smaller Fermi level shift upon doping.

## Discussion

### Effect of PH_3_ Plasma on Sputter Deposition of Cu_3–*x*_P

Five important roles of PH_3_ in the sputter deposition of Cu_3–*x*_P can be identified. First, PH_3_ is a source of P that enables growth of Cu_3–*x*_P from a metallic Cu target. Second, the presence of PH_3_ counteracts the tendency of Cu_3–*x*_P to decompose at elevated temperatures, enabling relatively high-temperature deposition where large crystal grains, *c*-axis texturing, and a higher V_Cu_ concentration can be achieved. Third, a very broad range of Cu/P ratios can be obtained at room temperature (Figure 5a), where P losses are minor. This possibility has allowed us to grow polycrystalline CuP_2_ films by a two-step process.^12^ Fourth, PH_3_ assists the crystallization of Cu_3–*x*_P even at room temperature, where films sputtered in pure Ar are amorphous (Figure 2). This effect is presumably caused by bombardment of the growing film by energetic species formed from the dissociation of PH_3_ in an RF plasma.^47^ Fifth, the net concentration of electrically active defects can be tuned by adjusting the PH_3_ partial pressure, independently of film composition (Figure 5c). This effect is a reminder that equilibrium defect concentrations ultimately depend on chemical potentials during growth, and not directly on film composition. The beneficial features listed in this section demonstrate that reactive RF sputtering is a highly versatile route for both fundamental studies of new phosphides and their technological development.

### Structural–Electrical Property Relationships

**Figure 9:**
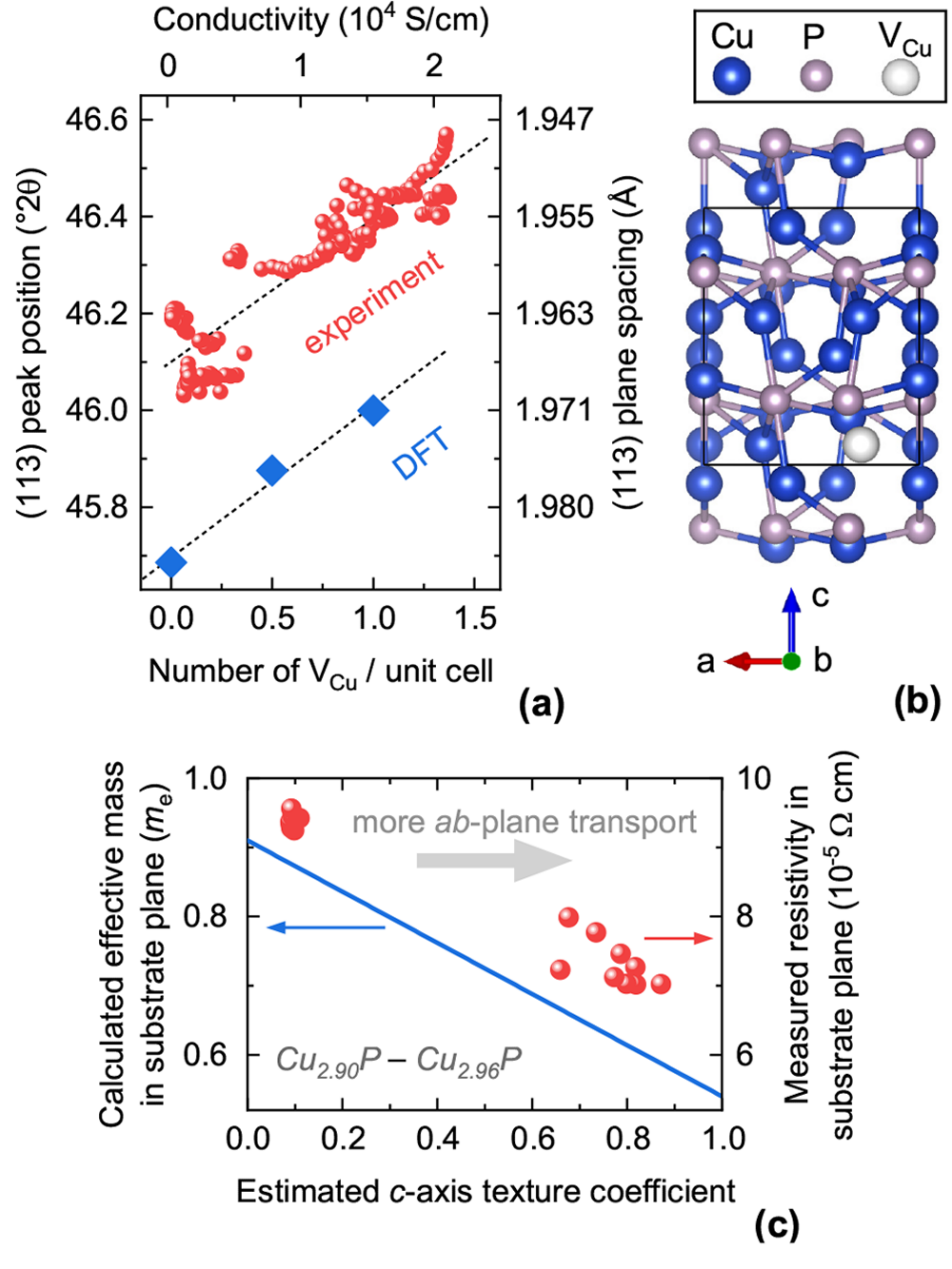
Relationships between structural and electrical properties in Cu_3–*x*_P films. (a) Spacing between (113) planes (determined from XRD peak position) as a function of measured conductivity. Also shown is the DFT-calculated (113) plane spacing for the case of zero, half, and one Cu vacancy per 24-atom unit cell. The conductivity axis and the V_Cu_ concentration axis are linked by assuming a constant mobility of 29 cm^2^/(V s) (Figure 6b). (b) The *P*6_3_*cm* crystal structure of Cu_3_P.^73^ The unit cell and the energetically preferred site for V_Cu_ are shown. (c) Experimentally measured resistivity as a function of the estimated *c*-axis texture coefficient. A coefficient of one indicates that the resistivity in the *ab*-plane is probed. A coefficient of zero indicates that the resistivity in a *c*-axis-containing plane is probed. Only films deposited at 220 °C with 2.90 < Cu/P < 2.96 are considered. The average conductivity effective mass of Cu_3–*x*_P in the probed transport plane is also plotted versus the texture coefficient, showing that the changes in resistivity can be explained by changes in the effective mass with lattice direction.

In Figure 4, we identified large variations in lattice constants and preferential orientation in Cu_3–*x*_P films processed under different conditions. Here, we investigate possible relationships between these structural trends and the electrical properties of the films. In general, the lattice constants of Cu_3–*x*_P films tend to decrease with increasing electrical conductivity. This behavior is shown in Figure 9a by measuring the (113) plane spacing in combinatorial samples with different conductivities. As noted earlier in this article, changes in the interplanar spacing in other directions indicate an overall unit cell contraction or expansion in all directions, rather than strain in one particular direction.

A simple explanation for this phenomenon is that the unit cell contracts when Cu vacancies are formed. Unit cell contraction is expected since the atoms surrounding the Cu vacancy can be packed closer together due to the free space left by the missing Cu atom. Very limited charge transfer is expected between Cu and P, due to the semimetallic (rather than ionic) nature of Cu_3–*x*_P. Hence, the counteracting effect of anion–anion repulsion, which may lead to unit cell expansion, is likely negligible. To verify this behavior, we employed DFT to calculate the equilibrium lattice constants of Cu_3–*x*_P in the presence of zero, half, and one Cu vacancy per unit cell. The Cu vacancy was placed in one of the symmetry-equivalent Cu(1) lattice sites following Olofsson’s notation.^13^ This site is indicated in Figure 9b and was previously found to be the most energetically favorable site for V_Cu_.^26^ As expected, the structure relaxes to lower lattice constants with increasing V_Cu_ concentration. For one vacancy per unit cell, the *a*, *b*, and *c* axes decrease by 0.56%, 0.65%, and 0.63% with respect to the intrinsic cell, respectively. The resulting (113) plane spacing for the calculated structures is shown in Figure 9a as a function of V_Cu_ concentration. The rate of change in the interplanar spacing is in quantitative agreement with the experimental behavior. The overall overestimation of lattice constants is a known problem of the PBE functional used for the relaxation.^74^ This result substantiates the hypotheses that (i) changes in V_Cu_ concentration are the main source of conductivity changes in Cu_3–*x*_P films and (ii) the estimate of 1–1.5 V_Cu_ per unit cell for the most electrically conductive films is reasonable.

Clearly, the decrease in lattice constants with increasing conductivity is only a rough experimental trend. Other factors such as defect compensation and variations in mobility are likely to affect the conductivity of a given film. For example, we may expect different hole mobilities μ in the *ab*-plane and along the *c*-axis, since the *P*6_3_*cm* structure of Cu_3–*x*_P is uniaxially anisotropic (Figure 9b). Under a p-type doping density of 3 × 10^21^ cm^–3^ at room temperature, we find a conductivity effective mass of 0.54 *m_e_* in the *ab*-plane and of 1.28 *m_e_* along the *c*-axis by applying Boltzmann transport theory. Assuming a direction-independent carrier scattering time τ, one would expect the resistivity ρ to be proportional to the effective mass m* in the transport direction, due to the ρ ∝ μ^–1^ ∝ m*τ^–1^ relationships. To test this hypothesis, we consider the Cu_3–*x*_P samples deposited at 220 °C. They were grown in the same deposition run, have similar composition (Cu_2.90_P–Cu_2.96_P), but have large differences in orientation with respect to the substrate depending on the prevalence of Cu_3–*x*_P formation at the target or at the substrate (Figure S3). Indeed, we find that the resistivity measured in the plane of the substrate is proportional to the average effective mass in the plane of the substrate, derived from the estimated texture coefficient (Figure 9c). Assuming that the changes in resistivity are caused by changes in hole mobility, this correlation provides experimental evidence for enhanced hole mobility in the *ab* plane. It is also an indirect indication that the carrier scattering time at room temperature is not strongly direction-dependent.

### Consequences of Nonstoichiometry

We have seen that the net concentration of acceptor defects increases with deposition temperature (Figures 5b and 8d) and PH_3_ partial pressure (Figure 5c). We have also gathered substantial evidence that these acceptors are Cu vacancies. However, an apparent discrepancy requires further discussion. A large fraction of films are expected to have a V_Cu_ concentration between 1 and 1.5 per unit cell (Figures 6a and 9a). These concentrations would correspond to Cu/P ratios between 2.75 and 2.83 in films without secondary phases or compensating defects. Yet, the most P-rich composition obtained in low-resistivity films is Cu_2.87_P. This compositional discrepancy is only 4% and may well be due to a small systematic error in the evaluation of elemental composition (see the Supporting Information). However, we also find that the electrical resistivity and the overall composition in the 2.95 < Cu/P < 3.05 range are completely uncorrelated (Figure 5c). This finding cannot be explained by measurement errors, because we estimate the error of relative composition measurements to be lower than 1% (more details in the Supporting Information).

A possible explanation is the existence of metallic Cu secondary phases for Cu/P > 2.75, so even in highly P-rich films. As discussed in the Supporting Information, this mechanism is incompatible with other experimental observations in this study. The presence of other point defects beyond Cu vacancies could be an alternative explanation for the composition-independent resistivity around the stoichiometric point. As discussed in the Supporting Information, different types of defects could potentially explain the experimental results—either extrinsic impurities or native defects and either donor- or charge-neutral defects. Some defects that would be compatible with our results are Cu_P_ (either donors or neutrals), H_i_ donors, or H_Cu_ neutrals. Hydrogen-related defects may result from H incorporation in a reactive film deposition process involving PH_3_ such as ours.^75^ A more extensive qualitative discussion of possible defects is given in the Supporting Information.

Besides point defects, there are other mechanisms that may change the overall film composition without changing the net dopant concentration. They are intermediate between formation of isolated point defects and segregation of secondary phases. One mechanism is the formation of Cu-rich donor–acceptor defect clusters such as (V_Cu_ + Cu_P_). Another possible mechanism is extended clustering driven by entropy.^76^ A dedicated study of defect energetics in Cu_3–*x*_P may help clarify which mechanism is dominant.

### On the Previous Identification of Cu_3–*x*_P as a Semiconductor

As mentioned earlier in this article, many authors have identified Cu_3–*x*_P as a degenerately doped semiconductor, rather than a (semi)metal.^16,24,26,28−30,33^ In many of these studies, the characterized samples were Cu_3–*x*_P nanoparticles or nanoplatelets with a characteristic size of less than 10 nm. In these cases, it is possible that size effects (quantum confinement) result in the opening of a band gap in an otherwise (semi)metallic system. A similar effect is known for 1*T*-TiS_2_.^77^ However, the results of our study also demonstrate that many criteria previously used to assign semiconducting behavior to Cu_3–*x*_P are not at all incompatible with a semimetal.

For example, several studies identified Cu_3–*x*_P as a semiconductor with a band gap in the 0.8 eV–1.7 eV range.^16,28,30,33^ This assignment was justified by the observation of an absorption onset in this photon energy range. However, Figure 8e demonstrates that the photon energy of the absorption onset is not related to a band gap, but it depends instead on the energy at which interband absorption becomes dominant over intraband (free-carrier) absorption. The photon energy of this apparent “band gap” ultimately depends on the free carrier density in the semimetal. If we remove the source of free carrier absorption (intraband transitions), we see that the absorption coefficient of Cu_3–*x*_P only approaches zero at zero photon energy, indicating that its band gap is zero (inset of Figure 8e, green dashed line).

In other cases, identification of Cu_3–*x*_P as a semiconductor was based on the detection of a positive Seebeck coefficient^26^ or of a photovoltaic effect.^24^ However, Boltzmann transport theory predicts positive Seebeck coefficients in semimetallic Cu_3_P, both under intrinsic- and p-type-doped conditions (Figure 7a). Many elemental metals also have positive Seebeck coefficients.^62^ A photovoltaic effect was observed in a Cu_3–*x*_P/CdS heterojunction.^24^ However, CdS is itself a photovoltaic material, and the reported photovoltaic parameters are compatible with a CdS-based solar cell in which Cu_3–*x*_P acts as a contact. In particular, the reported short-current density of 2.7 mA/cm^2^ is well within the Shockley–Queisser limit^78^ of 7.5 mA/cm^2^ for a CdS cell assuming a 2.4 eV band gap for CdS.^79^ In fact, Cu_3–*x*_P has recently been incorporated into a ZnSnP_2_ solar cell^22^ but rather as a hole contact than as an absorber.

Finally, one computational study found a small—but nonzero—band gap in pristine Cu_3_P by first-principles calculations using hybrid exchange correlation functionals and a Gaussian-type atomic orbital basis set.^80^ A band gap of 0.68 eV was obtained with the PBE0 functional, and a 0.18 eV gap was obtained with the HSE06 functional. However, unusually large values of the on-site Hubbard repulsion (*U*) were necessary to reproduce these band gaps by the DFT+U approach^81^ using a plane wave basis set. It is also unusual that Hubbard repulsion had to be added on the P 3p orbitals rather than just on the Cu 3d orbtals. Most importantly, the complex dielectric function calculated from the DFT+U band structure in the same study^80^ is incompatible with our present experimental results. As an example, the constant value of the calculated ε_1_ (around 4) up to 4 eV photon energy^80^ is in stark contrast with the high dispersion of the experimental ε_1_ (Figure 8a) in this spectral region (negative, up to 15, and negative again).

To summarize this section, there is no conclusive evidence in favor of identification of Cu_3–*x*_P as a semiconductor without invoking quantum confinement effects.

## Conclusion

We found strong evidence that reactively sputtered Cu_3–*x*_P films are natively doped p-type semimetals over a broad composition and process parameter range. Unlike the case of nonreactive sputtering, Cu_3–*x*_P films could be synthesized at temperatures significantly higher than 200 °C, where a higher density of native acceptors can be stabilized and, therefore, the p-type conductivity can be higher. We achieved room-temperature electrical resistivities down to 4.6 × 10^–5^ Ω cm, the lowest reported for Cu_3–*x*_P samples in any form.

In films deposited at temperatures above 370 °C, the single-phase region of Cu_3–*x*_P is likely not wider than the Cu_2.9_P–Cu_3.0_P composition range. However, we found some indications that polycrystalline Cu_3–*x*_P films deposited at room temperature may tolerate a higher deviation from stoichiometry on the Cu-rich side (*x* < 0).

The decreasing resistivity with increasing PH_3_ partial pressure, the preference for Cu-deficient compositions, and the unit cell contraction with decreasing resistivity substantiated the hypothesis that Cu vacancies are responsible for p-type conductivity in Cu_3–*x*_P. P-type conductivity was retained even when Cu/P > 3, indicating that Cu vacancies are more stable than any compensating donor even under Cu-rich conditions. Interestingly, stoichiometric defect-free Cu_3_P is also predicted to be a semimetal by DFT, though with a much lower carrier concentration and a net excess of electrons rather than holes. This “intrinsic” Cu_3_P may be very challenging to synthesize due to the high stability of Cu vacancies under all process conditions.

Despite the very high concentration of native dopants, the high mobility of Cu_3–*x*_P films at low temperatures (276 cm^2^/(V s) at 10 K) points to surprisingly low ionized impurity scattering rates. By studying transport properties as a function of lattice direction, we observed anisotropy in the electrical conductivity of Cu_3–*x*_P, with higher conductivities achieved along the *ab*-plane. This trend is in quantitative agreement with the expected inverse proportionality between hole effective masses (higher in the *c*-axis direction) and hole mobility.

A NIR absorption feature somewhat similar to the previously identified LSPR peak of Cu_3–*x*_P nanoparticles was found. Curiously, DFT calculations indicate that the interband transitions of intrinsic bulk Cu_3–*x*_P also give rise to an absorption peak in that spectral region. In thin-film samples, interband transitions are a more likely explanation for the experimentally observed peak, rather than a plasmonic response. Finally, Fermi level lowering in increasingly doped Cu_3–*x*_P films gave rise to an optical effect equivalent to the Burstein–Moss shift. From this effect, the carrier concentration can in principle be estimated from a simple optical transmission measurement.

## Supporting Information Available

Extended experimental and computational details, additional SEM images, Raman spectra, Seebeck coefficient and dielectric function measurements, extended discussion of secondary phases, Hall effect measurements, carrier concentration, mobility, and defects (PDF)
