# Supplementary material for: Is Cu3–xP a Semiconductor, a Metal, or a Semimetal?
Source: Chem Mater. 2023 Jan 25;35(3):1259–72. doi: 10.1021/acs.chemmater.2c03283 (PMC9933438; doi:10.1021/acs.chemmater.2c03283)
Supplement: Supplementary file 1 — cm2c03283_si_001.pdf [file cm2c03283_si_001.pdf]

SUPPORTING INFORMATION

Is $Cu_{3-x}P$ a semiconductor, a metal, or a semimetal?

Andrea Crovetto,[*,†,‡,¶] Thomas Unold,[‡] and Andriy Zakutayev[*,†]

†*Materials Science Center, National Renewable Energy Laboratory, Golden, Colorado 80401, United States*

‡*Department of Structure and Dynamics of Energy Materials, Helmholtz-Zentrum Berlin für Materialien und Energie GmbH, 14109 Berlin, Germany*

¶*National Centre for Nano Fabrication and Characterization (DTU Nanolab), Technical University of Denmark, 2800 Kongens Lyngby, Denmark*

E-mail: ancro@dtu.dk; andriy.zakutayev@nrel.gov

Experimental details

Film growth

Amorphous and polycrystalline $Cu_{3-x}P$ thin films were deposited by radio-frequency (RF) sputtering, either by non-reactive sputtering of a $Cu_{3-x}P$ target in pure Ar, or by reactive co-sputtering of a $Cu_{3-x}P$ target and a Cu target in PH_3/Ar, with PH_3 concentration up to 5%. The sputter system (PVD Products) had a base pressure in the 10^{-7} Torr range. The Cu target (K. J. Lesker Company) and the $Cu_{3-x}P$ target (Princeton Scientific) were 2″ in diameter, 0.25″ in thickness, and 99.99% pure. The two targets were co-sputtered at 5 mTorr total pressure, with RF powers of 40 W ($Cu_{3-x}P$ target) and 20 W (Cu target). The target-substrate distance was 16 cm and the deposition rate was about $0.7\,\text{Å}\,\text{s}^{-1}$. The deposition temperature was measured at the metallic platen onto which the substrates were clamped during deposition.

In each deposition process, a film was simultaneously grown on two Corning Eagle XG borosilicate glass substrates placed next to each other, covering a total area of $10 \times 5\,\text{cm}^2$. The targets were oriented so that one of the short edges of the total substrate area would mainly be coated by the Cu target and the other short edge by the $Cu_{3-x}P$ target. In this way, it was possible to obtain combinatorial gradients in film composition (Cu/P ratio) approximately parallel to the long edge. This combinatorial effect is evident in Fig. 1(b) of the main article. Each data point, spectrum, and XRD pattern in the main article corresponds to one specific point in the combinatorial films, which has its unique composition and properties. When data from a single measurement point is shown, the point comes from a film location approximately halfway between the $Cu_{3-x}P$ and the Cu target, unless otherwise specified.

The combinatorial data was managed with the COMBIgor tool,[1] the Research Data Infrastructure,[2] and was integrated into the High-Throughput Experimental Materials Database.[3] The thickness of each film varied by about 10–15% across the long direction (10 cm long). The thickness of all films considered in this study was between 200 nm and 300 nm.

Film characterization

Elemental composition and film thickness were determined by x-ray fluorescence (XRF) in a Bruker Tornado M4 instrument at 15 Torr pressure using a Rh source. XRF spectra were fitted with the Bruker XMethod analysis program. The XRF data was calibrated by Rutherford backscattering spectroscopy (RBS) measurements of separate Cu-P films of different thicknesses and compositions deposited on Si. These RBS measurements and the calibration were described in a previous publication.[4] The accuracy of RBS in determining composition is around 3%.[5] However, there are other possible sources of error. One of them is

the extra calibration step required to obtain composition information from XRF spectra. The other is the use of silicon substrates (rather than the glass substrates used for the rest of the characterization) for the calibration. Taking these factors into account, we estimate an overall *systematic* error in the $\pm 5\%$ range for the Cu/P ratios quoted in the main article. We expect the *random* error in the determination of composition by XRF to be much lower than 5%. The total x-ray counts in XRF measurements were sufficiently high to ensure better than 1% reproducibility. Variations in film thickness could be a source of "random" error in the extracted composition. However, the films have rather similar thicknesses and there were no noticeable correlations between thickness and the extracted Cu/P ratios. Finally, a systematic error in the $\pm 5\%$ range can be assumed for the thickness determined by XRF. This error comes from uncertainty in modeling surface roughness when fitting RBS spectra, from film porosity (resulting in lower density than in ideal Cu_3P) and from the XRF calibration step. The main consequence of this error is that it propagates to the resistivity. Hence, we also estimate a systematic error in the $\pm 5\%$ range for resistivity, Hall carrier concentration, and Hall mobility.

XRD measurements were conducted with a Bruker D8 diffractometer using Cu K_α radiation and a 2D detector. To cover the desired 2θ range, two frames were collected with the incidence angle ω fixed at 10° and 22.5°, and the detector center fixed at 2θ values of 35° and 60°, respectively. The diffraction intensity at each 2θ angle was integrated over the χ range measured by the 2D detector. Since reflections from a range of 2θ angles are measured in parallel by the 2D detector, this XRD measurement is not strictly in the Bragg-Brentano configuration and the lattice planes probed by XRD are not strictly parallel to the substrate plane. However, the angles between the probed lattice planes and the substrate plane are rather small (in the 0°–19° range depending on the value of 2θ with respect to the detector's center). Thus, we make the approximation that the lattice planes corresponding to XRD reflections are parallel to the plane of the substrate.

The c-axis texture coefficient (TC) was estimated as

$$TC = \frac{\frac{I(113)}{I_0(113)}}{\frac{I(113)}{I_0(113)} + \frac{I(300)}{I_0(300)}} \tag{S1}$$

where $I(113)$ and $I(300)$ are the integrated intensities of the (113) and (300) peaks in the thin film, and $I_0(113)$ and $I_0(300)$ are the integrated intensities of the (113) and (300) peaks in a randomly oriented $Cu_{3-x}P$ powder, taken from Olofsson's work.[6] With this definition, a film with the (113) planes perfectly parallel to the substrate has a texture coefficient of 1, and a film with (300) planes perfectly parallel to the substrate has a texture coefficient of 0. Note that the [001] direction, rather than the [113] direction, corresponds to the c-axis of the lattice. The reason for using the (113) peak rather than the (002) peak at $\sim 24.9°$ 2θ for

texture analysis is that the (002) reflection is very weak and not detectable in many samples (see Fig. 2 in the main article). Conversely, the (113) reflections are detected in all samples and form a relatively small angle (18°) with the ideal (001) planes. Considering the two approximations described above, our derived texture coefficient should only be taken as a semi-quantitative estimation, so it is labeled "estimated texture coefficient" in the figures of the main article. Nevertheless, the conclusions drawn in the article on the basis of the texture coefficient are also semi-quantitative. Hence, our approximations do not affect the high-level conclusions on preferential orientation (Fig. 4(b) of the main article) and direction-dependent conductivity (Fig. 9(c) of the main article).

Raman spectra were measured with a Renishaw inVia Raman microscope under laser light excitation at 532 nm wavelength with a 100× objective. Scanning electron microscopy (SEM) images were taken with a Hitachi S-3400N instrument with a field emission gun and 5 kV beam voltage.

Sheet resistance was measured in the substrate plane with a collinear four-point probe directly contacting the film. The sheet resistance was derived by multiplying the raw ohmic resistance by $\pi/\ln(2)$, as appropriate for the collinear geometry. The electrical resistivity was derived by multiplying the sheet resistance by the XRF-determined thickness. Temperature-dependent Hall carrier concentration and mobility were measured in the substrate plane with a Lake Shore 8425 DC Hall System using the van der Pauw configuration. The $Cu_{3-x}P$ film employed for this measurement was deposited through a shadow mask to obtain a Hall cross shape, and Ti/Pt contacts were evaporated at the edges of the cross. The DC driving current and magnetic fields were 1 mA and 2 T respectively. The Hall voltage at each temperature was determined as the average of 8 measurements, by reversing sign of the current and of the magnetic field, and by considering two non-equivalent contact geometries. The Seebeck coefficient of an unpatterned sample with about the same electrical resistivity was measured in a custom-built setup using In contacts and four temperature differences in the vicinity of room temperature.

The complex dielectric function was extracted by spectroscopic ellipsometry using a J.A. Woollam M-2000 ellipsometer and three incidence angles. We modeled the system as a glass substrate of known optical functions, a $Cu_{3-x}P$ layer of unknown optical functions, and a roughness layer treated with Bruggeman effective medium theory. The optical functions of $Cu_{3-x}P$ were represented by a Kramers-Kronig-consistent b-spline function with 0.1 nodes/eV. The samples analyzed in this study were too absorbing and too thick for ellipsometry to yield thickness information (no light reflected from the film/glass interface). Hence, we fixed the $Cu_{3-x}P$ thickness to the XRF-measured value in the ellipsometry model. Ellipsometry spectra were fitted with the CompleteEase software (J.A. Woollam). The absorption coefficient was derived from the complex dielectric function using standard optical relations. The absorbance shown in Fig. 8(c) of the main article was derived by measuring transmission T at normal incidence and reflection R at near-normal

incidence with a Cary 7000 spectrophotometer. The measurement was performed with an integrating sphere to include the diffuse component of both transmission and reflection. The absorbance A was extracted as

$$A = -\log_{10}\left(\frac{T}{(1-R)}\right) \tag{S2}$$

Computational details

Density functional theory calculations

First-principles calculations were performed using Density Functional Theory (DFT) within the Projector-Augmented Wave (PAW) formalism[7] and a plane-wave basis set as implemented in the GPAW code,[8,9] in combination with the Atomic Simulation Environment (ASE).[10] The Perdew-Burke-Ernzerhof (PBE) exchange correlation functional[11] was employed for structural relaxation. The plane-wave cutoff and **k**-mesh density were 450 eV and 8×8×4, respectively. The structures were relaxed until the forces were less than 0.05 eV/Å. For the calculations on $Cu_{3-x}P$ with one Cu vacancy/unit cell, the vacancy was introduced at one of the symmetry-equivalent Cu(1) sites (one of the two inequivalent sites at 6c Wyckoff positions), according to the notation of Olofsson.[6] For the calculations on $Cu_{3-x}P$ with half a Cu vacancy/unit cell, a 2×1×1 (48-atom) supercell was constructed with a Cu vacancy at one of the Cu(1) sites.

Ground-state electronic structure calculations were performed with the GLLB-SC exchange correlation functional[12,13] with a plane-wave cutoff of 450 eV and a **k**-mesh density of 16×16×8. Kramers-Kronig-consistent dielectric function spectra were calculated by linear response theory within the Random Phase Approximation (RPA) including local field effects, as implemented in GPAW. The absorption coefficient was derived from the dielectric function using standard relations.

Semiclassical transport calculations

Temperature- and doping density-dependent transport properties of $Cu_{3-x}P$ were estimated using Boltzmann transport theory as implemented in BoltzTraP2.[14] The input of the calculations was an interpolation of the previously calculated PBE band structure of Cu_3P available on the Materials Project database (mp-7463).[15,16] The BoltzTraP2 code was interfaced with Materials Project through the pymatgen package.[17,18] A constant carrier scattering time of 10 fs was assumed for all bands at all energies. The effective masses quoted in the main article are the eigenvalues of the conductivity effective mass tensor, calculated at a temperature of 300 K under a net p-type doping density of $3 \times 10^{21}\,\mathrm{cm}^{-3}$. Assuming a hole mobility μ of $29\,\mathrm{cm}^2/\mathrm{Vs}$ as measured by Hall effect (Fig. 6(b), main article), a 10 fs scattering time τ corresponds to a hole

effective mass m^* of 0.61 m_e using the relation $\mu = e\tau/m^*$. This value is close to the calculated effective mass of 0.54 m_e in the ab-plane of doped Cu_3P at room temperature. These considerations justify the choice of the carrier scattering time.

The effective mass as a function of texture coefficient TC was estimated by assuming that the a-axis is always lying on the substrate plane, that the component of the b-axis vector parallel to the substrate plane is $|b|(TC)$, and that the component of the c-axis vector parallel to the substrate plane is $|c|(1-TC)$. With this description, there are two relevant hole effective masses in the substrate plane (which is the transport plane probed by the resistivity measurement). One is $m_a^* = 0.54\ m_e$ and the other is $m_{b/c}^* = (TC)m_b^* + (1-TC)m_c^*$, where $m_a^* = m_b^*$ due to symmetry, and $m_c^* = 1.28\ m_e$ is the c-axis effective mass. The overall effective mass in the substrate plane was simply taken as the arithmetic average between m_a^* and $m_{b/c}^*$.

Supplementary discussion

Cu secondary phases in $Cu_{3-x}P$ films

From the experimental data in Fig. 1(b) of the main article, we note that the range of Cu-rich (Cu/P > 3) compositions free of metallic Cu peaks becomes narrower with increasing PH_3 partial pressure. This observation can be explained as follows. A substrate reaction (phosphorization of metallic Cu) is dominant at substrate locations closer to the Cu target. Direct transport of $Cu_{3-x}P$ vapor from the target is dominant at substrate locations closer to the $Cu_{3-x}P$ target. As the PH_3 partial pressure increases, a given Cu/P ratio in the films is obtained at locations closer and closer to the Cu target. Thus, the fraction of the incoming vapor consisting of metallic Cu rather than already-formed $Cu_{3-x}P$ increases. Since an additional reaction at the substrate is necessary to form $Cu_{3-x}P$ from metallic Cu, the segregation of Cu as a polycrystalline impurity is more likely.

Interpretation of Hall carrier concentrations in $Cu_{3-x}P$

Semiclassical transport simulations have revealed that there can be a major discrepancy between the net carrier concentration $(p - n)$ in $Cu_{3-x}P$ and the carrier concentration n_H derived from a Hall measurement assuming a single carrier type. The reason is the comparable concentrations of electrons and holes in $Cu_{3-x}P$ near its intrinsic Fermi level. The offset between the two quantities is illustrated in Fig. 7(b) in the main article. Because of this discrepancy, the values of n_H as a function of Fermi level predicted by Boltzmann transport theory can be counterintuitive. Here we list some potentially counterintuitive features.

1. The calculated $|n_H|$ is always above $1.5 \times 10^{21}\,\mathrm{cm}^{-3}$ regardless of the Fermi level position (Fig. 7(b) in the main article). Hence, we expect that a Hall effect measurement on a $Cu_{3-x}P$ film will always indicate very high carrier concentrations n_H, even in a hypothetical film with a very low net carrier concentration.

2. n_H diverges at the Fermi level when the numerator of R_H approaches zero and changes sign (about 10 meV above the intrinsic Fermi level).

3. The Fermi level where n_H changes sign is ∼70 meV higher than the Fermi level of zero net carrier concentration. This implies that there is a ∼70 meV Fermi level range where Cu_3P would appear as p-type from a Hall measurement (positive n_H), even though electrons are more abundant than holes. Intrinsic, defect-free Cu_3P is one of such cases ($p - n < 0$ but $n_H > 0$).

4. If $S_h/S_e \simeq \mu_h/\mu_e$, the numerators of Eq. 1 and Eq. 2 in the main article are zero at about the same net

carrier concentration. Thus, intrinsic, defect-free Cu_3P would also appear as p-type in a thermovoltage measurement (positive Seebeck coefficient).

Temperature dependence of carrier concentration in $Cu_{3-x}P$ films

The Hall hole concentration of a $Cu_{3-x}P$ film decreases by roughly a factor two with decreasing temperature from 300 K to 10 K (Fig. 6(a) in the main article). A small decrease in Hall hole concentration with temperature is also predicted by Boltzmann transport theory on Cu_3P with a Fermi level at 0.3 eV below the intrinsic value (Fig. 6(a), main article). However, the theoretically predicted decrease is much smaller than the experimental one. Possible reasons for this discrepancy could be: (i) entropy-driven formation of additional Cu vacancies with increasing temperature, as quantified before 30; (ii) band structure modifications occurring at such a high defect concentrations; (iii) temperature-driven structural changes accompanied by a rearrangement of Cu vacancies, as previously reported;[19] and (iv) a decrease in the Hall scattering factor r with decreasing temperature. When a single type of carrier is dominant, the Hall carrier concentration is defined as $n_H = r/eR_H$, where R_H is the Hall coefficient, e is the elementary charge, and the Hall scattering factor r is assumed to be equal to 1.[20] The Hall scattering factor depends on the energy dependence of the carrier scattering time (taken as a constant in our Boltzmann transport calculations). Thus, a decrease in Hall hole concentration by a factor 2 from 300 K to 10 K could be explained by a decrease in Hall scattering factor in the same temperature range. A decreasing Hall scattering factor with decreasing temperature occurs, for example, in various metals[21] and in graphene.[22]

Mobility of $Cu_{3-x}P$ films in comparison with other thin-film materials with similar doping levels

The room-temperature mobility of a $Cu_{3-x}P$ film (28.8 cm^2/Vs) is not unusual for non-epitaxial polycrystalline thin-film materials with carrier concentrations above 10^{21} cm^{-3}, such as transparent conductive oxides and elemental metals. However, the mobility of 276 cm^2/Vs measured at 10 K is particularly high. Due to the high hole concentration in $Cu_{3-x}P$, we expect ionized impurity scattering to dominate over grain boundary scattering. We estimated the ionized impurity scattering-limited mobility as $\mu_i = 279$ cm^2/Vs by fitting temperature-dependent mobility data (Fig. 6(b) of the main article). Here, we compile values of the Hall mobility measured in non-epitaxial polycrystalline thin-film materials with carrier concentrations comparable to $Cu_{3-x}P$.

In the chemically-related compound CaCuP – a p-type semiconductor degenerately doped by Cu vacancies and also deposited by RF reactive sputtering – μ_i was only 45.2 cm^2/Vs.[23] This is despite the fact that the

hole concentration of CaCuP was over an order of magnitude lower than in $Cu_{3-x}P$ and that its room-temperature mobility was slightly higher (36.4 cm^2/Vs).

In the heavily-doped (n-type) transparent conductive oxides In_2O_3:Sn (ITO) and ZnO:Al (AZO), μ_i was in the 10 cm^2/Vs–50 cm^2/Vs range, even though their carrier concentration was lower, in the 10^{20} cm^{-3} range.[24]

Non-epitaxial, n-type films of the doped narrow-gap semiconductors PbTe and Bi_2Te_3 deposited by RF sputtering had mobilities of 15 cm^2/Vs–40 cm^2/Vs at room temperature, and these mobilities decreased with decreasing temperature.

The low-temperature mobility of $Cu_{3-x}P$ films is even higher than the corresponding mobility of non-epitaxial polycrystalline films of elemental metals. For example, mobilities around 100 cm^2/Vs were reported for evaporated Cu and Au films on glass at 77 K measurement temperature.[25] The mobility of $Cu_{3-x}P$ films at the same temperature is about 180 cm^2/Vs (Fig. 6(b) of the main article). The mobility measurements on metals were conducted on sufficiently thick films (>100 nm), in which the mobility was not negatively affected by the limited thickness. Note that Cu and Au have more than an order of magnitude higher carrier concentrations than $Cu_{3-x}P$, but their carriers are intrinsic instead of being provided by defects.

We emphasize, however, that both elemental metals and doped narrow-gap semiconductors can have much higher low-temperature mobilities (well above 10^3 cm^2/Vs) if grown epitaxially or as single crystals.

Possible causes of composition-independent resistivity of $Cu_{3-x}P$ films near Cu/P = 3

The electrical resistivity and the overall composition of $Cu_{3-x}P$ films in the $2.95 < Cu/P < 3.05$ range are completely uncorrelated (Fig. 5(c), main article). The conceptually simplest explanation for a composition-independent resistivity is the existence of metallic Cu secondary phases for Cu/P > 2.75, so even in highly P-rich films. This hypothesis can be summarized as follows: (i) the films consist of a $Cu_{3-x}P$ phase and a Cu phase regardless of the Cu/P ratio; (ii) the point defects present in the $Cu_{3-x}P$ phase are Cu vacancies; (iii) the composition of the $Cu_{3-x}P$ phase is dictated by the V_{Cu} concentration but *not* by the overall Cu/P ratio, so for example when 1 V_{Cu}/unit cell, the composition of the $Cu_{3-x}P$ phase is $Cu_{2.83}P$; (iv) the observed variations in the *overall* composition are caused by varying concentrations of metallic Cu phases depending on process conditions.

A problem with this hypothesis is that we have indeed observed metallic Cu by SEM, but only in films with Cu/P > 3.0 (Fig. S2). Another problem is that a film with overall $Cu_{3.00}P$ composition doped with 1.5 V_{Cu}/unit cell would need to contain 6.5% metallic Cu by volume, if Cu secondary phases were fully responsible for deviations in the expected stoichiometry. However, the fraction of the top surface of a

$Cu_{3.00}P$ film that is covered by secondary phases visible in the SEM can be estimated as being only 0.6% (Fig. S2(b)). Unless metallic Cu preferentially segregates at the bottom of the film, it seems unlikely that all the Cu in excess of the expected $Cu_{3-x}P$ composition exists in the form of a secondary phase.

A second possible reason for a composition-independent resistivity near Cu/P = 3 is the existence of additional point defects beyond Cu vacancies. Different types of defects could be plausible candidates for explaining the composition-independent resistivity – either extrinsic impurities or native defects, and either compensating donors or charge-neutral defects. Regardless of the type of defect, the variations in composition near Cu/P = 3 are unlikely to be linked to different chemical potentials of Cu and P during growth. If the two quantities were linked, the resistivity should depend on composition, since we have ascertained that changing the chemical potentials by other means (i.e., different PH_3 partial pressures) does lead to changes in resistivity (Fig. 5(c), main article).

Native defects

The Cu_P antisite could be a possible candidate. In the related material CaCuP, defect calculations have shown that Cu_P can be in the singly or doubly ionized state (donor), or in the neutral state.[23] If Cu_P exists in the singly ionized state in $Cu_{3-x}P$, it may compensate the formation of additional V_{Cu} acceptors beyond a certain V_{Cu} concentration threshold. Formation of one Cu_P donor for each V_{Cu} acceptor would then keep the resistivity constant, and it would increase the overall Cu/P ratio with increasing defect compensation (one less P atom per pair of compensating defects). Interestingly, in this scenario the Cu/P ratio would *increase* with increasing V_{Cu} concentration at high defect concentrations.

Even if Cu_P is in the neutral state rather than in the ionized state, it could still be a possible candidate for a composition-independent resistivity. With Cu_P in the neutral state, an increasing concentration of Cu_P defects would increase the overall Cu/P ratio (one extra Cu atom and one less P atom per defect) at constant V_{Cu} concentration (constant resistivity).

V_P donors could, in principle, be another possible compensating defect that would drive the overall composition towards higher Cu/P ratios. However, compensating V_P donors would likely result in decreasing unit cell volume with increasing compensation. This would lead to more scattering in the conductivity versus lattice constant data (Fig. 9(a)) at high conductivities, which is not observed. Cu interstitials (Cu_i) in the neutral state could also modulate the Cu/P ratio at constant V_{Cu} concentration. However, increasing Cu_i concentrations are likely to lead to increasing unit cell volumes. Thus, neither V_P donors nor Cu_i neutrals are likely to cause the composition-independent resistivity of $Cu_{3-x}P$ films near the stoichiometric point.

Extrinsic defects

No impurities heavier than Na could be detected in XRF spectra of $Cu_{3-x}P$ films. Among lighter elements, the only two impurities that may be present in $Cu_{3-x}P$ films at atomic concentrations above 1% are oxygen (from imperfect vacuum in the deposition chamber) and hydrogen (from PH_3 decomposition). A small oxygen peak was detected in energy-dispersive x-ray (EDX) spectra of most of our $Cu_{3-x}P$ samples, with overall O concentration estimated at below 2 at.%. Hydrogen content in $Cu_{3-x}P$ films could not be measured with the techniques available to us. A previous study on Zn_3P_2 films deposited by reactive sputtering at 150 °C in a PH_3-containing atmosphere reported a H content of 4 at.% using secondary ion mass spectrometry (SIMS).[26] We expect a lower – but not insignificant – hydrogen content in our $Cu_{3-x}P$ films due to the higher deposition temperature (370 °C), which usually facilitates hydrogen desorption.

Hence, both H and O are likely to be present at low but not negligible concentrations in most our $Cu_{3-x}P$ films. In the case that H and O are incorporated in $Cu_{3-x}P$ as point defects, we hypothesize that they are most likely to form interstitials (H_i and O_i). The reason is their small size compared to both Cu and P, as estimated by their covalent radii.[27] As an exception, the H_{Cu} antisite may also be energetically favorable, due to the high availability of vacant Cu sites in $Cu_{3-x}P$.

H_i is typically found to be a compensating donor in p-type materials.[28] Thus, it is not implausible that H_i donors may compensate V_{Cu} acceptors above a certain V_{Cu} concentration threshold. In this scenario, the decrease in Cu/P ratio from 3.05 to 2.90 in Fig. 5(c) of the main article may be due to an increase in V_{Cu} concentration, which is however compensated by H_i formation leading to a constant resistivity. In this case, the decrease in unit cell size by formation of additional V_{Cu} defects may be counteracted by an increase in unit cell size due to an equal concentration of interstitials. A change in Cu/P ratio from 3.05 to 2.90 due to increasing V_{Cu} concentration would require H concentrations in the film around 4 at.%, assuming that all H impurities act as H_i defects. As mentioned above, it cannot be excluded that our films have a H impurity concentration in this range. O_i is rarely found to be a donor in other materials, so it is less likely to explain our experimental results.

If H_{Cu} is a charge-neutral defect (as may be expected from oxidation state arguments), changes in H_{Cu} concentration at constant V_{Cu} concentration could also explain the composition-independent resistivity close to Cu/P = 3. In this scenario, the V_{Cu} concentration would be constant as a function of overall film composition (keeping the resistivity constant), but the Cu/P would decrease as the concentration of H_{Cu} defects increases.

Supplementary figures

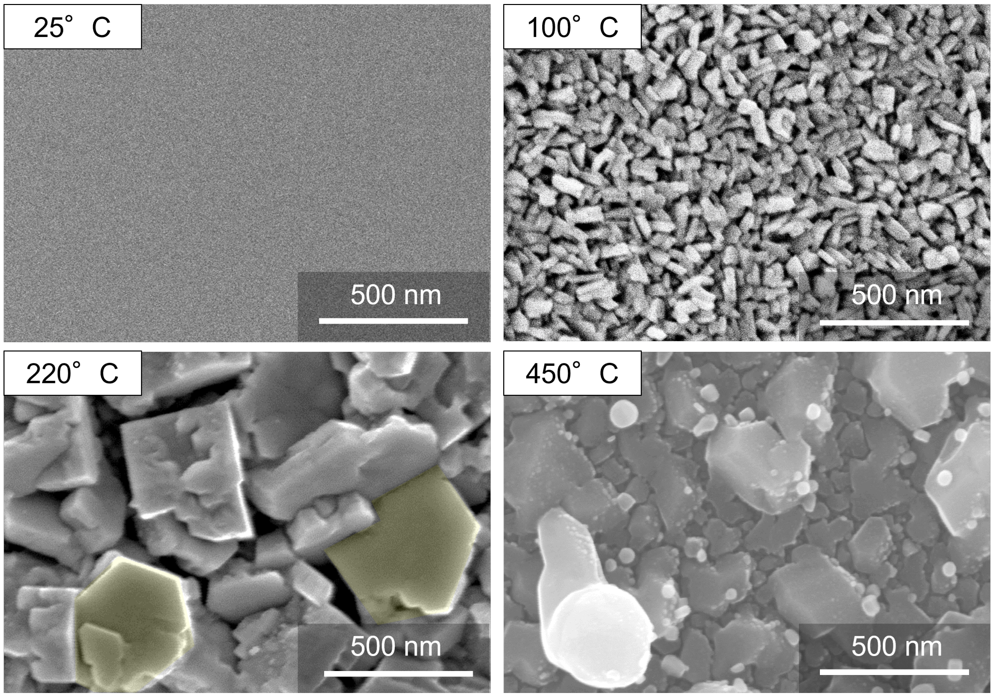

Figure S1: SEM images of $Cu_{3-x}P$ films sputter-deposited in pure Ar at different substrate temperatures. The films deposited at 25 °C, 100 °C, and 220 °C have composition close to Cu_3P. The film deposited at 450 °C has composition $Cu_{6.45}P$ due to phosphorus evaporation from the growing film. Most crystal grains in the film deposited at 220 °C have hexagonal shapes (highlighted in yellow), in line with the hexagonal symmetry of the $P6_3cm$ crystal structure of $Cu_{3-x}P$.

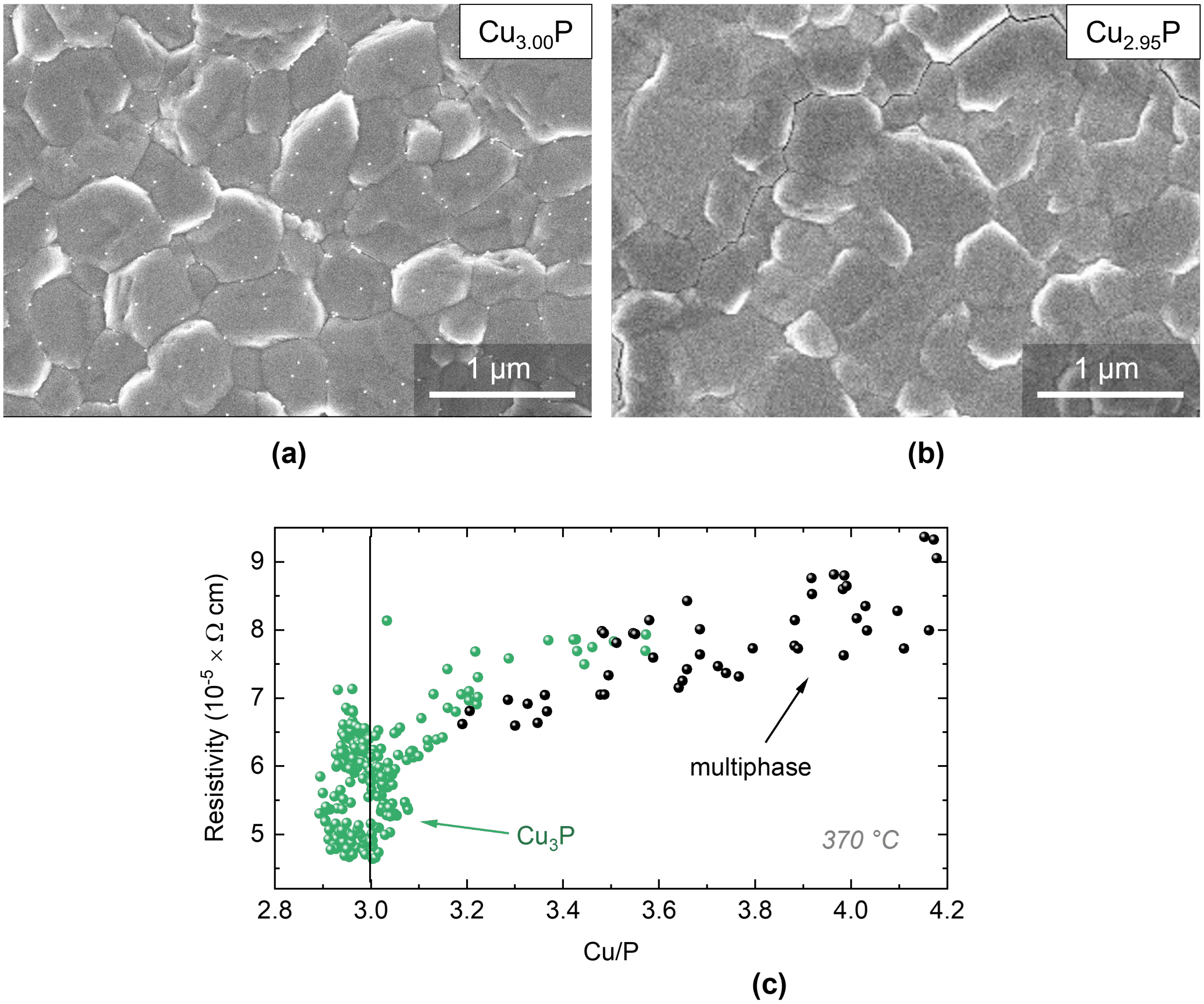

Figure S2: **(a):** SEM image of a film with overall $Cu_{3.00}P$ composition, where bright secondary phases are observed throughout the film surface. The diameter of the secondary phases is roughly 10 nm. The area coverage of the secondary phases is roughly 0.6%. Since metallic Cu is detected by XRD at slightly Cu-richer compositions, the phases observed in the SEM are also likely to consist of metallic Cu. This is compatible with their brighter appearance in the SEM, usually a sign of higher average atomic number. **(b):** SEM image of a film with overall $Cu_{2.95}P$ composition, where bright secondary phases are not observed. The films in (a) and (b) were deposited at 530 °C. **(c):** Composition-dependent resistivity of $Cu_{3-x}P$ films deposited at 370 °C. Samples in which no polycrystalline secondary phases were detected by XRD are shown in green. Samples which exhibited both $Cu_{3-x}P$ peaks and metallic Cu peaks in their XRD patterns are shown in black. Note that the presence or absence of polycrystalline Cu does not significantly affect the resistivity of films in the $3.2 < Cu/P < 3.6$ range. This finding confirms that amorphous Cu is very likely to exist in the $3.2 < Cu/P < 3.6$ range when polycrystalline Cu is not detected.

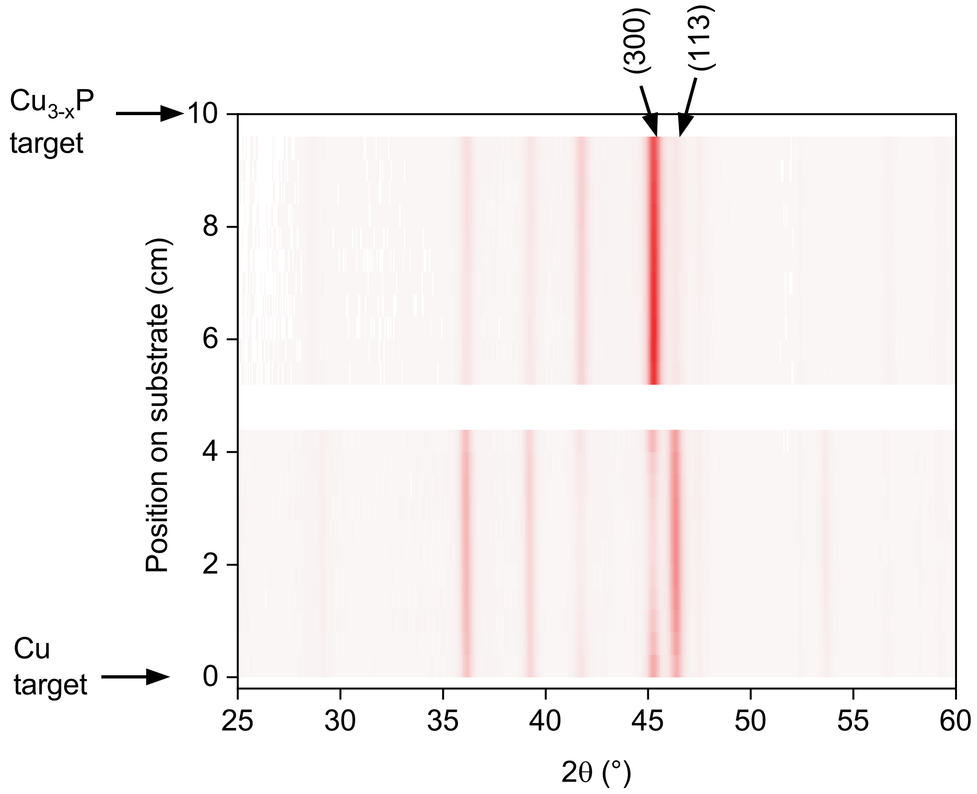

Figure S3: Color map representing XRD intensity from a combinatorial $Cu_{3-x}P$ film deposited at 220 °C as a function of position on the substrate with respect to the two sputter targets. At 0 cm position, the substrate was mainly coated by the metallic Cu target. At 10 cm position, the substrate was mainly coated by the $Cu_{3-x}P$ target. This dataset was used for orientation-dependent resistivity measurements because of its wide range of texture coefficients, arising from the different intensity ratios between the (300) peak and the (113) peak at different positions. The positions closer to the $Cu_{3-x}P$ target have a particularly strong (300) texture, resulting in a very low c-axis texture coefficient of 0.1 (Fig. 4(b), main article). This property is unique in our whole dataset of $Cu_{3-x}P$ samples. It may be related to a transition growth zone that is only accessible within a relatively narrow parameter space.[29] Indeed, SEM images of $Cu_{3-x}P$ films deposited from a $Cu_{3-x}P$ target in pure Ar at 220 °C (Fig. S1) reveal a unique morphology (hexagonally faceted grains) not observed under other deposition conditions.

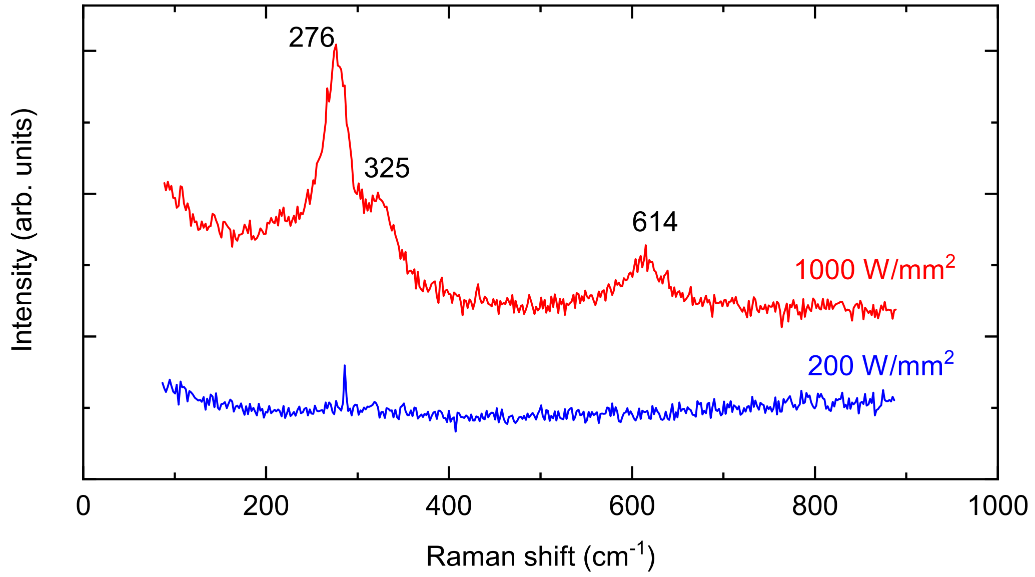

Figure S4: Raman spectra of a $Cu_{2.95}P$ film deposited at 370 °C. No peaks are observed at an excitation intensity of 200 W/mm^2, which is very high for Raman spectroscopy, but still slightly below the ablation threshold of the film. The absence of Raman peaks in $Cu_{3-x}P$ is compatible with some of the existing literature.[30,31] Three peaks compatible with other previous studies[32–34] appear at an excitation intensity of 1000 W/mm^2. However, this extremely high excitation intensity leads to film ablation, as clearly seen by optical microscopy after the Raman measurement.

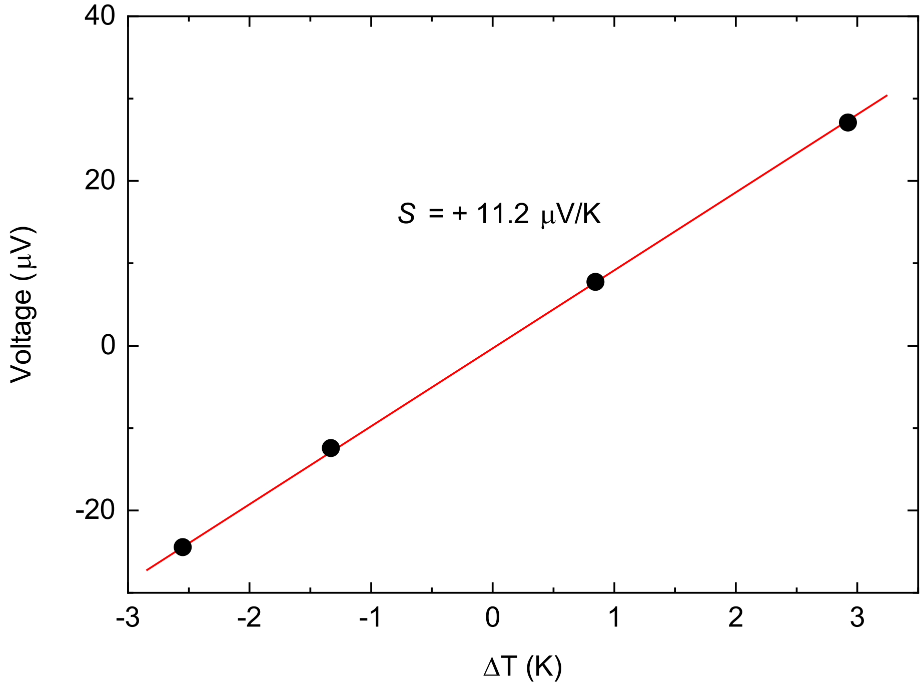

Figure S5: Thermovoltage measurements on a $Cu_{2.95}P$ film grown in the same way as the sample used for Hall effect measurements (Fig. 6 of the main article) and with similar electrical resistivity. Two corners of the film were contacted with In wire and one contact was heated to create a temperature gradient. The temperature difference and the voltage between the two contacts were measured after temperature stabilization. The Seebeck coefficient S of $Cu_{2.95}P$ is extracted as the slope of the thermovoltage versus temperature difference curve, plus the known Seebeck coefficient of In. The positive sign of S confirms that holes are majority charge carriers.

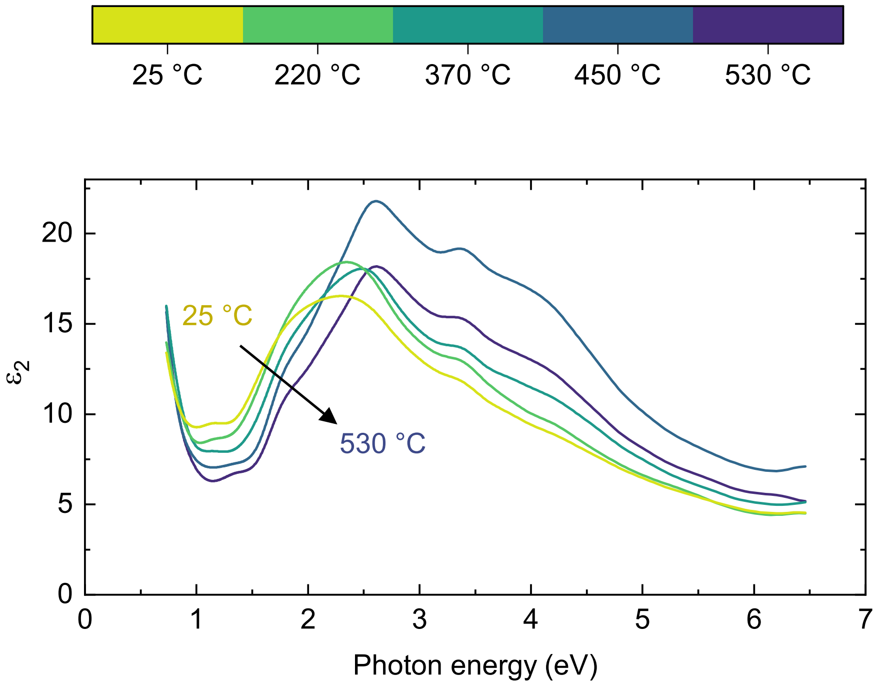

Figure S6: Ellipsometry-measured imaginary part of the dielectric function (ε_2) for the lowest-resistivity sample at each deposition temperature. The position of the maximum occurring between 2 eV and 3 eV photon energy is used to plot Fig. 8(d) in the main article. The blue-shift of this maximum, and the blue-shift of the onset of the increase in ε_2 around 1.5 eV, are indications of a Burstein-Moss effect caused by the increasing hole concentration with increasing deposition temperature.

References

(1) Talley, K. R.; Bauers, S. R.; Melamed, C. L.; Papac, M. C.; Heinselman, K. N.; Khan, I.; Roberts, D. M.; Jacobson, V.; Mis, A.; Brennecka, G. L.; Perkins, J. D.; Zakutayev, A. COMBIgor: Data-Analysis Package for Combinatorial Materials Science. *ACS Combinatorial Science* **2019**, *21*, 537–547.

(2) Talley, K. R.; White, R.; Wunder, N.; Eash, M.; Schwarting, M.; Evenson, D.; Perkins, J. D.; Tumas, W.; Munch, K.; Phillips, C.; Zakutayev, A. Research Data Infrastructure for High-Throughput Experimental Materials Science. *Patterns* **2021**, *2*, 100373.

(3) Zakutayev, A.; Wunder, N.; Schwarting, M.; Perkins, J. D.; White, R.; Munch, K.; Tumas, W.; Phillips, C. An Open Experimental Database for Exploring Inorganic Materials. *Scientific Data* **2018**, *5*, 180053.

(4) Crovetto, A.; Kojda, D.; Yi, F.; Heinselman, K. N.; LaVan, D. A.; Habicht, K.; Unold, T.; Zakutayev, A. Crystallize It before It Diffuses: Kinetic Stabilization of Thin-Film Phosphorus-Rich Semiconductor CuP_2. *Journal of the American Chemical Society* **2022**, *144*, 13334–13343.

(5) Verma, H. R. *Atomic and Nuclear Analytical Methods: XRF, Mössbauer, XPS, NAA and Ion-Beam Spectroscopic Techniques*; Springer: Berlin Heidelberg, 2007.

(6) Olofsson, O. The Crystal Structure of Cu_3P. *Acta Chemica Scandinava* **1972**, *26*, 2777–2787.

(7) Blöchl, P. E. Projector Augmented-Wave Method. *Physical Review B* **1994**, *50*, 17953–17979.

(8) Mortensen, J.; Hansen, L.; Jacobsen, K. Real-Space Grid Implementation of the Projector Augmented Wave Method. *Physical Review B* **2005**, *71*, 035109.

(9) Enkovaara, J. et al. Electronic Structure Calculations with GPAW: A Real-Space Implementation of the Projector Augmented-Wave Method. *Journal of Physics: Condensed Matter* **2010**, *22*, 253202.

(10) Larsen, A. H. et al. The Atomic Simulation Environment—a Python Library for Working with Atoms. *Journal of Physics: Condensed Matter* **2017**, *29*, 273002.

(11) Perdew, J. P.; Burke, K.; Ernzerhof, M. Generalized Gradient Approximation Made Simple. *Physical Review Letters* **1996**, *77*, 3865–3868.

(12) Kuisma, M.; Ojanen, J.; Enkovaara, J.; Rantala, T. T. Kohn-Sham Potential with Discontinuity for Band Gap Materials. *Physical Review B* **2010**, *82*, 115106.

(13) Gritsenko, O.; van Leeuwen, R.; van Lenthe, E.; Baerends, E. J. Self-Consistent Approximation to the Kohn-Sham Exchange Potential. *Physical Review A* **1995**, *51*, 1944–1954.

(14) Madsen, G. K.; Carrete, J.; Verstraete, M. J. BoltzTraP2, a Program for Interpolating Band Structures and Calculating Semi-Classical Transport Coefficients. *Computer Physics Communications* **2018**, *231*, 140–145.

(15) Jain, A.; Ong, S. P.; Hautier, G.; Chen, W.; Richards, W. D.; Dacek, S.; Cholia, S.; Gunter, D.; Skinner, D.; Ceder, G.; Persson, K. A. Commentary: The Materials Project: A Materials Genome Approach to Accelerating Materials Innovation. *APL Materials* **2013**, *1*, 011002.

(16) Ong, S. P.; Cholia, S.; Jain, A.; Brafman, M.; Gunter, D.; Ceder, G.; Persson, K. A. The Materials Application Programming Interface (API): A Simple, Flexible and Efficient API for Materials Data Based on REpresentational State Transfer (REST) Principles. *Computational Materials Science* **2015**, *97*, 209–215.

(17) Ong, S. P.; Richards, W. D.; Jain, A.; Hautier, G.; Kocher, M.; Cholia, S.; Gunter, D.; Chevrier, V. L.; Persson, K. A.; Ceder, G. Python Materials Genomics (Pymatgen): A Robust, Open-Source Python Library for Materials Analysis. *Computational Materials Science* **2013**, *68*, 314–319.

(18) Ricci, F.; Chen, W.; Aydemir, U.; Snyder, G. J.; Rignanese, G.-M.; Jain, A.; Hautier, G. An Ab Initio Electronic Transport Database for Inorganic Materials. *Scientific Data* **2017**, *4*, 170085.

(19) Wolff, A. et al. Low-Temperature Tailoring of Copper-Deficient $Cu_{3-x}P$ —Electric Properties, Phase Transitions, and Performance in Lithium-Ion Batteries. *Chemistry of Materials* **2018**, *30*, 7111–7123.

(20) Werner, F. Hall Measurements on Low-Mobility Thin Films. *Journal of Applied Physics* **2017**, *122*, 135306.

(21) Alderson, J. E. A.; Farrell, T.; Hurd, C. M. Hall Coefficients of Cu, Ag, and Au in the Range 4.2-300°K. *Physical Review* **1968**, *174*, 729–736.

(22) Macheda, F.; Poncé, S.; Giustino, F.; Bonini, N. Theory and Computation of Hall Scattering Factor in Graphene. *Nano Letters* **2020**, *20*, 8861–8865.

(23) Willis, J.; Bravić, I.; Schnepf, R. R.; Heinselman, K. N.; Monserrat, B.; Unold, T.; Zakutayev, A.; Scanlon, D. O.; Crovetto, A. Prediction and Realisation of High Mobility and Degenerate P-Type Conductivity in CaCuP Thin Films. *Chemical Science* **2022**, *13*, 5872–5883.

(24) Ellmer, K.; Mientus, R. Carrier Transport in Polycrystalline ITO and ZnO:Al II: The Influence of Grain Barriers and Boundaries. *Thin Solid Films* **2008**, *516*, 5829–5835.

(25) Chopra, K. L.; Bahl, S. K. Hall Effect in Thin Metal Films. *Journal of Applied Physics* **1967**, *38*, 3607–3610.

(26) Weber, A.; Sutter, P.; von Känel, H. Optical, Electrical, and Photoelectrical Properties of Sputtered Thin Amorphous Zn_3P_2 Films. *Journal of Applied Physics* **1994**, *75*, 7448–7455.

(27) Pyykkö, P.; Atsumi, M. Molecular Single-Bond Covalent Radii for Elements 1-118. *Chemistry - A European Journal* **2009**, *15*, 186–197.

(28) Van de Walle, C. G.; Neugebauer, J. Hydrogen in Semiconductors. *Annual Review of Materials Research* **2006**, *36*, 179–198.

(29) Thornton, J. A. High Rate Thick Film Growth. *Annual Review of Materials Science* **1977**, *7*, 239–260.

(30) De Trizio, L.; Gaspari, R.; Bertoni, G.; Kriegel, I.; Moretti, L.; Scotognella, F.; Maserati, L.; Zhang, Y.; Messina, G. C.; Prato, M.; Marras, S.; Cavalli, A.; Manna, L. $Cu_{3-x}P$ Nanocrystals as a Material Platform for Near-Infrared Plasmonics and Cation Exchange Reactions. *Chemistry of Materials* **2015**, *27*, 1120–1128.

(31) Hou, C.-C.; Chen, Q.-Q.; Wang, C.-J.; Liang, F.; Lin, Z.; Fu, W.-F.; Chen, Y. Self-Supported Cedarlike Semimetallic Cu_3P Nanoarrays as a 3D High-Performance Janus Electrode for Both Oxygen and Hydrogen Evolution under Basic Conditions. *ACS Applied Materials and Interfaces* **2016**, *8*, 23037–23048.

(32) Liu, S.; He, X.; Zhu, J.; Xu, L.; Tong, J. Cu_3P/RGO Nanocomposite as a New Anode for Lithium-Ion Batteries. *Scientific Reports* **2016**, *6*, 1–10.

(33) Peng, X.; Lv, Y.; Fu, L.; Chen, F.; Su, W.; Li, J.; Zhang, Q.; Zhao, S. Photoluminescence Properties of Cuprous Phosphide Prepared through Phosphating Copper with a Native Oxide Layer. *RSC Advances* **2021**, *11*, 34095–34100.

(34) Peng, X.; Lv, Y.; Zhao, S. Chemical Vapor Deposition and Thermal Oxidation of Cuprous Phosphide Nanofilm. *Coatings* **2022**, *12*, 68.
